# *Anopheles Gambiae* PRS1 Modulates *Plasmodium* Development at Both Midgut and Salivary Gland Steps

**DOI:** 10.1371/journal.pone.0011538

**Published:** 2010-07-12

**Authors:** Thomas Chertemps, Christian Mitri, Sylvie Perrot, Jean Sautereau, Jean-Claude Jacques, Isabelle Thiery, Catherine Bourgouin, Isabelle Rosinski-Chupin

**Affiliations:** 1 Unité de Biochimie et Biologie Moléculaire des Insectes, Département de Parasitologie et Mycologie, Centre National de la Recherche Scientifique URA 3012, Institut Pasteur, Paris, France; 2 CEPIA, Département de Parasitologie et Mycologie, Institut Pasteur, Paris, France; Université Pierre et Marie Curie, France

## Abstract

**Background:**

Invasion of the mosquito salivary glands by *Plasmodium* is a critical step for malaria transmission. From a SAGE analysis, we previously identified several genes whose expression in salivary glands was regulated coincident with sporozoite invasion of salivary glands. To get insights into the consequences of these salivary gland responses, here we have studied one of the genes, *PRS1* (*Plasmodium responsive salivary 1*), whose expression was upregulated in infected glands, using immunolocalization and functional inactivation approaches.

**Methodology/Principal Findings:**

*PRS1* belongs to a novel insect superfamily of genes encoding proteins with DM9 repeat motifs of uncharacterized function. We show that *PRS1* is induced in response to *Plasmodium*, not only in the salivary glands but also in the midgut, the other epithelial barrier that *Plasmodium* has to cross to develop in the mosquito. Furthermore, this induction is observed using either the rodent parasite *Plasmodium berghei* or the human pathogen *Plasmodium falciparum*. In the midgut, PRS1 overexpression is associated with a relocalization of the protein at the periphery of invaded cells. We also find that sporozoite invasion of salivary gland cells occurs sequentially and induces intra-cellular modifications that include an increase in *PRS1* expression and a relocalization of the corresponding protein into vesicle-like structures. Importantly, *PRS1* knockdown during the onset of midgut and salivary gland invasion demonstrates that *PRS1* acts as an agonist for the development of both parasite species in the two epithelia, highlighting shared vector/parasite interactions in both tissues.

**Conclusions/Significance:**

While providing insights into potential functions of DM9 proteins, our results reveal that PRS1 likely contributes to fundamental interactions between *Plasmodium* and mosquito epithelia, which do not depend on the specific *Anopheles*/*P. falciparum* coevolutionary history.

## Introduction

Malaria, one of the most devastating infectious diseases is caused by an Apicomplexa parasite of the genus *Plasmodium* whose transmission occurs through the bite of an infected *Anopheles* mosquito. In the mosquito vector, *Plasmodium* completes a complex developmental program involving a series of molecular and cellular interactions leading to the colonization of the salivary glands and the production of infectious sporozoites [1], [2], [3]. Understanding these finely controlled events is essential to designing new strategies to reduce malaria transmission.

Parasite development within the vector begins when the mosquito ingests an infective bloodmeal from a vertebrate host. After gamete fertilization inside the mosquito midgut lumen, the resulting zygote rapidly transforms into a motile ookinete that crosses the midgut epithelium and stops migrating when it makes contact with the midgut basal lamina. Here the ookinete gives rise to an oocyst that undergoes intensive internal mitotic divisions and yields up to several thousand sporozoites that are released into the mosquito body cavity. Sporozoites invade salivary glands by crossing the basal lamina and the plasma membrane of the salivary gland cell [4], [5]. Cell traversal is followed by storage inside a large extracellular secretory cavity. From here, sporozoites can access the salivary duct and be injected along with salivary proteins during the mosquito's bite, allowing transmission to the vertebrate host. A large increase in sporozoite infectivity towards the vertebrate host occurs during their storage in the salivary glands.

While both motile forms of the parasite, the ookinete and the sporozoite, must cross an epithelial barrier, respectively the midgut and the salivary gland, different interactions are anticipated in these processes. In the midgut, the ookinete first interacts with the latero-apical pole of the cell processes. In the midgut, the ookinete first interacts with the latero-apical pole of the cell; then, the ookinete gets access to the membranous labyrinth beneath the basal lamina through intra and intercellular routes. In some of the traversed cells, cell invasion elicits reactions leading to apoptosis or necrosis and extrusion from the epithelial layer. Large parasite losses occur as a consequence of host defensive reactions [6], [7], [8], [9]. In contrast, sporozoites first interact with the salivary gland basal lamina and then enter salivary cells through the basal membrane forming a transient vacuole. Although salivary glands are invaded by thousands of sporozoites, this process does not seem to be detrimental to the epithelium [4], [5], suggesting that efficient mechanisms of cell reparation have been developed.

Cellular responses during midgut invasion have been extensively studied over the last 5 years using different combinations of *Plasmodium* and *Anopheles*. Large-scale expression analyses have highlighted both classical humoral immune responses and more local epithelial responses [10], [11], [12]. These analyses also showed that the extent of the responses and the identity of the underlying genes are not fully conserved among the different parasite-vector combinations, possibly reflecting the degree of adaptation between parasite and mosquito. Additionally, extensive laboratory experiments mainly utilizing the *P. berghei* rodent malaria model have further explored the consequences of these systemic and local responses on ookinete survival and oocyst development establishing that the outcome of infection depends on finely balanced factors that affect parasite development in the mosquito both positively and negatively [10], [11], [12], [13], [14].

In contrast to numerous studies on parasite-host relationships in the midgut, very few studies have investigated sporozoite-salivary gland relationships including whether the two epithelia may share common fundamental reactions to parasite invasion, despite apparent differences in the invasion process. From a SAGE analysis, we identified 57 *Anopheles gambiae* genes differentially expressed in the salivary glands of infected female mosquitoes and showed that, as in the midgut, *Plasmodium* invasion is accompanied by an innate immune response as well as more general cellular responses [15]. Among the identified immune responsive genes, the *Serine Protease Inhibitor 6* (*SRPN6*) gene has recently been shown to negatively affect *P. berghei* sporozoite numbers in *An. gambiae* salivary glands [16].

Here we have conducted a functional analysis on PRS1, a protein containing DM9 motifs whose gene was identified by SAGE as upregulated during salivary gland invasion by Plasmodium [15]. This gene was found to be induced in response to *Plasmodium* invasion not only in the salivary glands but also in the midgut. We show that *PRS1* is a novel agonist of the *Plasmodium* developmental cycle in the two epithelia and exerts this function towards both *P. berghei* and *P. falciparum*. This suggests that, as previously demonstrated in the midgut, some important cellular mechanisms that benefit *Plasmodium* development in the salivary glands are not dependent on specific co-adaptations. Furthermore, this study reveals that cellular response pathways to *Plasmodium* invasion might be shared by the two major mosquito epithelial barriers.

## Results

### 1) PRS1 belongs to a novel protein family characterized by DM9 repeat motifs

**Figure 1:**
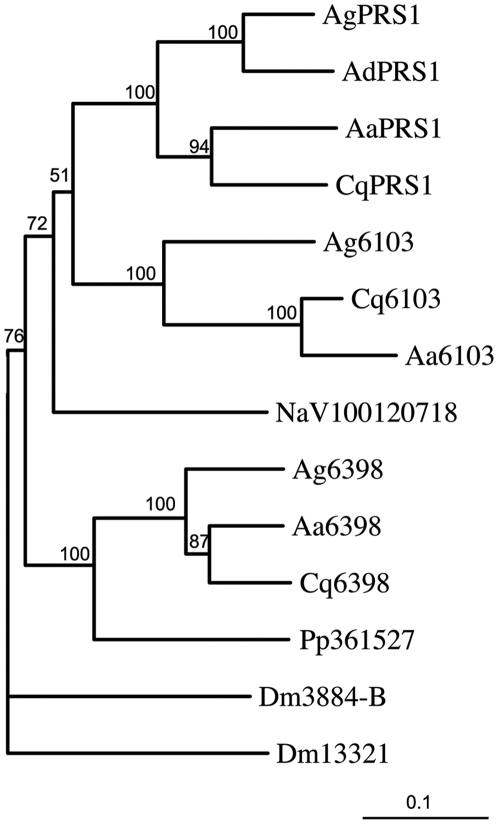
Partial phylogenetic tree of the DM9-protein family. DM9 proteins whose genes are located on chromosome 2 in *An. gambiae* were used for a blast search and the sequences of their closest homologues from different species: Ag, *An. gambiae*; Ad, *An. darlingi*; Cq, *Culex quinquefasciatus*; Aa, *A. aegypti*; Pp, *Phlebotomus papatasi*; NaV, *Nasonnia vitripennis* were aligned, together with two DM9 proteins from Drosophila (Dm: *D. melanogaster*). The sequence alignment was used to generate an unrooted tree Bootstrap values on 100 replicates are given. The scale bar represents 10% differences in protein sequences. The sequence alignment used to build the tree is shown in Figure S1, a more complete phylogenetic tree is provided in Figure S2 and a list of DM9 proteins with accession numbers is given in Table S1.

*PRS1* (*Plasmodium Responsive Salivary 1*) was previously described in a salivary gland transcriptome analysis [17] and was identified as upregulated in *An. gambiae* salivary glands upon invasion by *P. berghei* sporozoites [15]. The sequence of a corresponding full-length cDNA (BX037582, [18]) indicates that *PRS1* potentially encodes a 144 amino acid cytoplasmic protein, containing two DM9 motifs (smart00696). These motifs were first described in *Drosophila*, where they exist as repeat motifs, in association or not, with other conserved motifs (such as a Ring motif). Proteins with DM9 motifs are essentially found in arthropods and platyhelminths and only occasionally in other eukaryotes or prokaryotes (Table S1). In *An. gambiae*, five proteins, in addition to PRS1, display DM9 motifs. The corresponding genes are localized in three clusters on *An. gambiae* chromosomes. The first cluster of genes, comprising *AGAP009604*, *AGAP009605* and *AGAP009606*, is localized on the *An. gambiae* chromosome 3R. In AGAP009604, the DM9 motif is associated with a domain homologous to the farnesoic methyl transferases of crustaceans [19]. The two other clusters, respectively coding for PRS1 (AGAP006102) and AGAP006103 on the one hand, and AGAP006398 on the other hand, are found on chromosome 2L of the PEST genome, in the region of the 2La chromosomal inversion near the locus identified as being involved in the control of *Plasmodium* development [20]. This gene arrangement in clusters is suggestive of an evolution by gene duplication. Phylogenetic analysis reveals that *PRS1* and *AGAP006103* result from a relatively ancient gene duplication that probably occurred before Anophelinae and Culicinae separation (Fig. 1, S1 and S2). Both proteins share only 50% identical amino acids. Interestingly in PRS1 and AGAP006103, the DM9 motifs are not associated with any other sequence suggesting that they are the support for a putative biological activity.

### 2) *Plasmodium* invasion of mosquito midgut and salivary glands induces PRS1 expression

**Figure 2:**
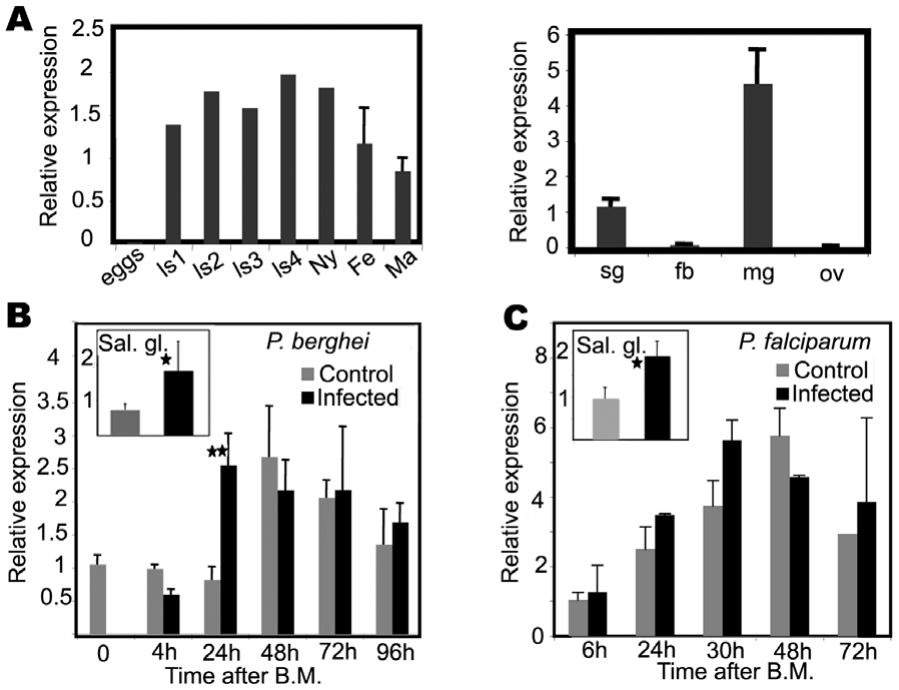
*PRS1* induction in salivary glands and the midgut after *P. berghei* or *P. falciparum* infection. A. qRT-PCR analysis of *PRS1* expression at different developmental stages and in different *Anopheles* tissues. Data were normalized to *An. gambiae* ribosomal protein *S7* mRNA levels. ls1-4, various instar larval stages; ny, nymphal stage; Fe, whole adult female; Ma, whole adult male; Sg, salivary glands; fb, fat body; mg, midgut; ov, ovary. Mean +/− SEM. B. qRT-PCR analysis of *PRS1* induction by *P. berghei* during midgut invasion by ookinetes (main panel) and salivary gland invasion (inset). Salivary glands were dissected 14 days after feeding on infected or control mice. The results are the mean +/− SEM of qPCR data normalized to *S7* expression obtained from at least three independent infections. Stars indicate significant differences; one star: p<0.05; 2 stars: p<0.001. C. qRT-PCR analysis of *PRS1* induction by *P. falciparum* during midgut invasion by ookinetes (main panel) and salivary gland invasion (inset). Salivary glands were dissected 11 days after feeding on gametocyte-positive (infected) or control (non-infected) blood. Results were obtained from two (midgut) or three (salivary glands) independent infections. Stars indicate significant differences; one star: p<0.05; 2 stars: p<0.001. No statistical analysis was conducted on midgut invasion by *P.falciparum* (only two replicates).

To characterize the expression pattern of *PRS1*, RT-PCR experiments were performed in various *Anopheles* tissues as well as during larval development (Fig. 2A). These experiments showed that, except at the egg stage, *PRS1* is expressed throughout the life of the mosquito, in males as well as in females. In addition to salivary glands, *PRS1* mRNA were detected in the midgut, indicating that *PRS1* is expressed in the two epithelia critical for *Plasmodium* development in *Anopheles*.

Since the midgut is the first epithelial barrier that *Plasmodium* has to pass to develop in the mosquito, we asked whether ookinete invasion might affect *PRS1* expression. To this end, *An. gambiae* was infected with *P. berghei* and *PRS1* expression monitored by qRT-PCR in midguts collected at different time points after infection. As shown in Fig. 2B, *PRS1* mRNA levels increased three-fold at 24 h after feeding on infected mice and were significantly higher (p<0.001) than in mosquitoes fed on non-infected mice. This induction is coincident with midgut invasion by ookinete. However, by 48 h both infected and uninfected samples showed the same increase in *PRS1* expression, probably as a secondary global response to bloodmeal (BM). A two- to three-fold increase in PRS1 mRNA levels was also observed in the salivary glands by 14 days on, after an infecting blood-meal, as previously described [15].

Next, we examined *PRS1* expression during invasion of midguts and salivary glands by the human pathogen, *P. falciparum*. As shown in Fig. 2C, *P*. *falciparum* also triggers overexpression of *PRS1* in the midgut by 24–30 h. and in the salivary glands by 11 days after the infecting BM, consistent with the kinetics of midgut and salivary gland invasion by this *Plasmodium* species. Therefore the response to both *P. berghei* and *P. falciparum* suggests that *PRS1* may be involved in *Plasmodium*/vector interactions regardless of the *Plasmodium* species.

### 3) Ookinete invasion induces PRS1 overexpression and relocalization in midgut cells

During traversal of the midgut epithelium, ookinetes elicit systemic and local immune responses leading to their recognition and destruction together with cellular responses such as cytoskeleton reorganization, cell protrusion and apoptotic cell death [6], [7]. To gain insight into the function of PRS1, we undertook its localization at the cellular level, using anti-PRS1 antibodies. These antibodies detected a protein of the expected molecular mass (16 kDa) in immunoblot analysis of midgut and salivary gland extracts from non-infected Anopheles (Figure S3A). In agreement with the transcript analysis, an increase in PRS1 levels occurred in the midgut 24 h after feeding on *P. berghei*-infected compared to non-infected mice (Figure S3B).

**Figure 3:**
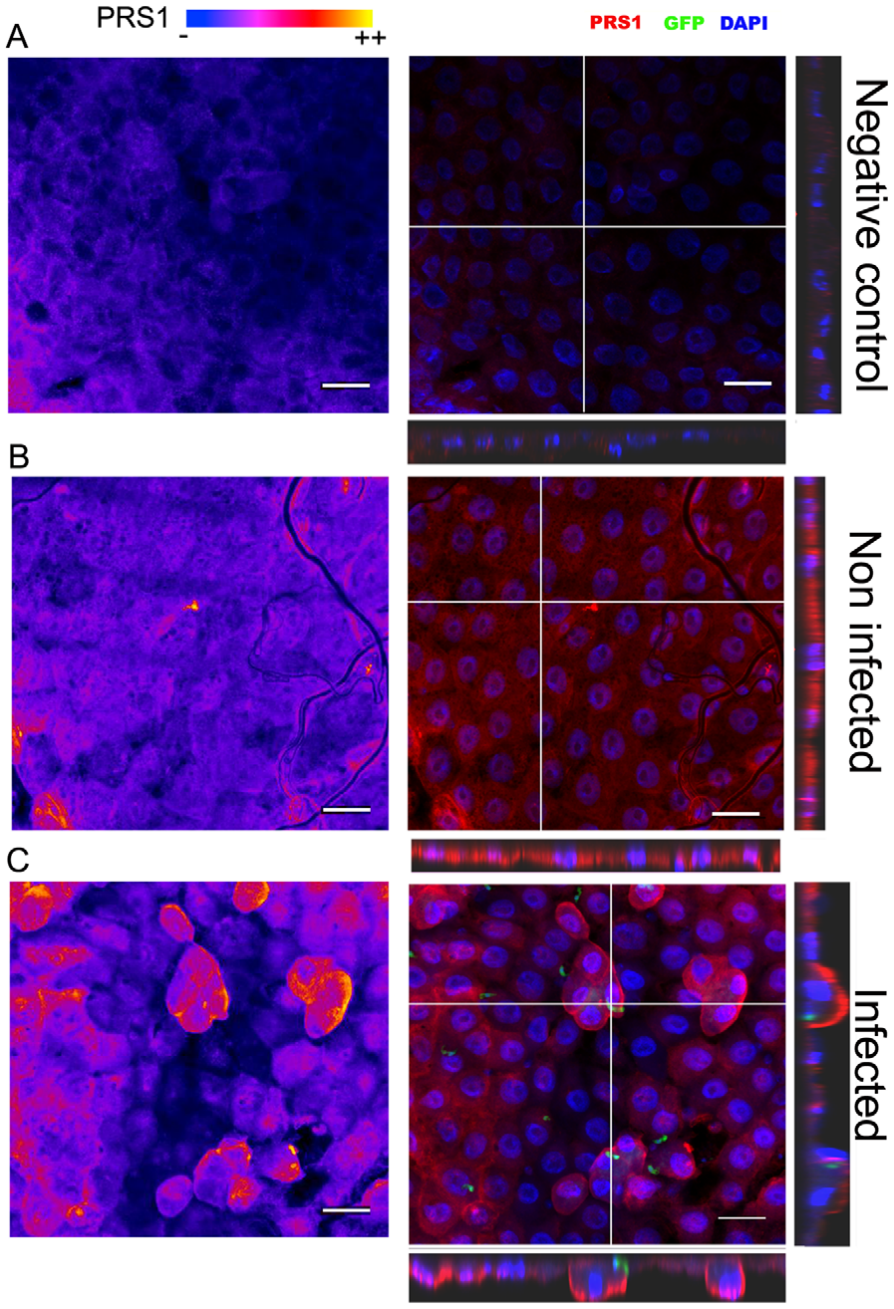
Confocal microscopy of triple-stained, infected and non-infected *A. gambiae* midguts. Midgut sections were incubated with preimmune serum (A) or anti-PRS1 immune serum (B and C) in the same way in parallel experiments and analyzed by confocal microscopy; the image acquisition parameters of the confocal microscope were maintained the same. In the left panels, Z-stack projections of PRS1 staining are shown in a false color representation (labeling intensity increasing from blue to yellow). In the right panels, a merge image of the three channels (red: PRS1; green: GFP-expressing ookinetes; blue: DAPI-counterstaining of the nuclei) is given according three planes: z-stack projection of the xy planes (main image), xz and yz cross-sections according to the directions marked by the white lanes on the main image. A: Control midgut stained with preimmune serum. B. Mosquito midgut, 24 h after a non-infective blood-meal. C. Mosquito midgut 24 h after feeding on a *P. berghei*-infected mouse. Note the higher intensity in PRS1 labeling in the proximity of ookinetes, as well as the relocalization of PRS1 in the cell periphery of cells protruding towards midgut lumen. Bar: 20 µm.

PRS1 localization during ookinete invasion of midgut cells was then investigated by immunofluorescence and confocal microscopy using anti-PRS1 antibodies and GFP-expressing *P. berghei*. The fluorescence intensity was compared between midgut sections prepared and analyzed in the same conditions. As observed in Fig. 3, the fluorescent labeling is higher on infected than non-infected midgut sections. To obtain a more precise quantification of this difference, the fluorescence intensity was estimated and found to be in mean 7-fold higher in infected midguts (relative fluorescence intensities compared to sections labeled with preimmune serum: 1.7 +/− 1.6 (non infected midgut sections); 13.6 +/− 10.3 (infected midgut sections). N = 3). In non-infected midguts, the anti-PRS1 labeling was relatively homogeneous (Fig. 3B). In contrast, in the *Plasmodium*-infected midguts, a more heterogeneous fluorescence pattern was detected (Fig. 3C), with 80% of the regions with higher fluorescence intensities found in the proximity of an ookinete. Furthermore, 90% of the ookinetes were detected near more intensely labeled cells, showing that the increase in PRS1 expression is associated with midgut cell invasion by the ookinetes.

In particular, PRS1 was overexpressed in isolated cells or in clusters of cells that protruded towards the midgut lumen (Fig. 3C). These cell protrusions were previously demonstrated to be linked to apoptotic cell death induced by invasion [6]. Consistently, most of these protruding cells contained or were in the proximity of an ookinete. Some rare protruding cells were not associated with a GFP- labeled ookinete. Since only live ookinetes express GFP [14], these cells might have been traversed by an ookinete that was subsequently killed by the immune response.

Interestingly, in the protruding cells, whatever their association with a live parasite, PRS1 was concentrated at the cell periphery, on the lumen size of the cell (Fig. 3C). Such a relocalization was also observed in some of the cells overexpressing PRS1 that were not protruding towards the midgut lumen. This was in contrast with a more widespread cellular distribution of PRS1 in the surrounding cells and suggested that PRS1 was relocalized inside the midgut cell in response to ookinete invasion and/or apoptotic cell death.

### 4) Salivary gland invasion is a sequential process marked by PRS1 relocalization in vesicle-like structures

PRS1 localization was then investigated in non-infected and *Plasmodium*-infected salivary gland cells, using GFP-expressing *P. berghei* sporozoites. To reveal potential cytoskeleton changes associated with Plasmodium invasion, we also labeled the actin network with fluorescent phalloidin.

**Figure 4:**
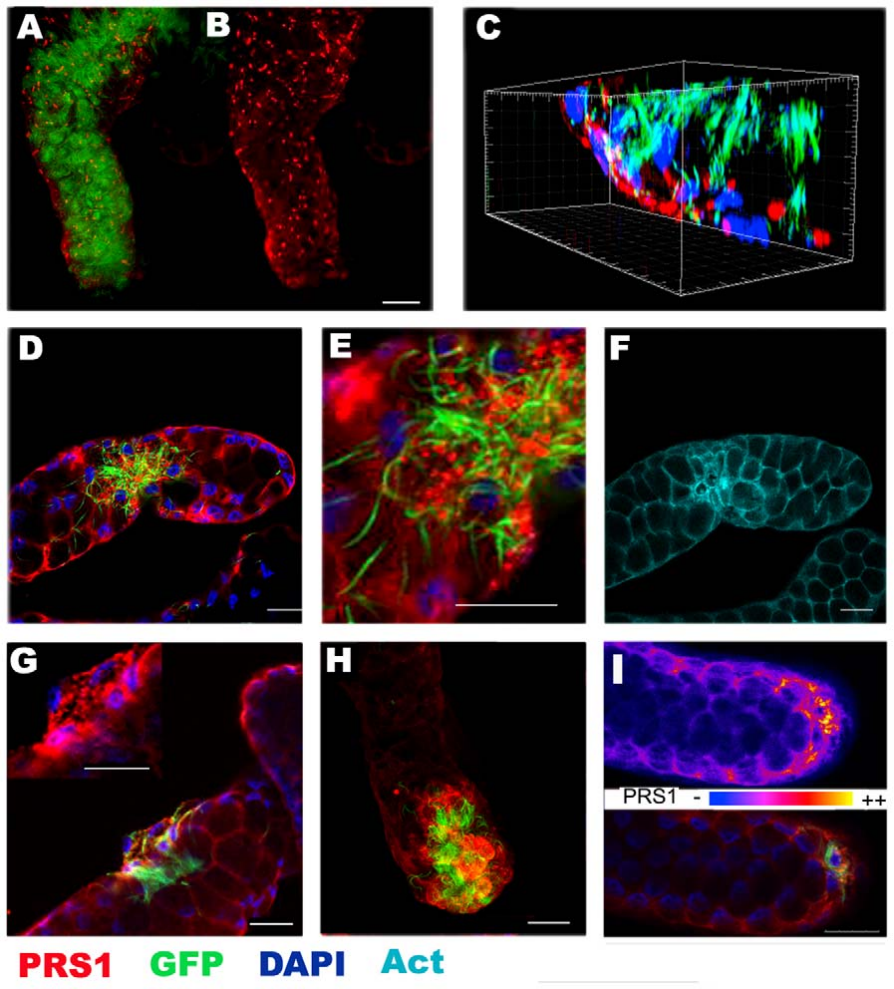
PRS1 overexpression in infected salivary glands. A–B. Z-stack projections of confocal sections for a highly infected salivary gland stained for PRS1 and GFP (A) or PRS1 alone (B).Note the granular pattern of PRS1 labeling. C. Three-dimension reconstruction of the gland shown in A/B, showing PRS1 localization in the basal (facing the hemolymph) side of the salivary cells. No PRS1 labeling can be observed in the central part of the gland. PRS1 labeling does not colocalize with sporozoite GFP-labeling. D–I. Confocal analysis of glands with localized sporozoite invasion. D: merge of PRS1, GFP and DAPI staining on a confocal section in a superficial plane of the gland. E: zoom view of the section observed in D. F: phalloidin labeling of the actin network for the same section as in D, revealing actin network reorganization in the invaded part of the gland. G: confocal section (middle plane; merge of PRS1, GFP and DAPI staining) of a gland characterized by a very localized invasion. PRS1 labeling in the infected zone at a higher magnification is given in the inset. H: Z-stack projection of confocal sections showing another example of localized infection, at the distal part of the salivary lobe. Note the higher intensity of PRS1 labeling in the infected part contrasting with the low level of labeling outside. I: PRS1 labeling in a false color representation (labeling intensity increasing from blue to yellow) (upper panel) and merge of PRS1, GFP and DAPI staining (lower panel) on a superficial section of a poorly infected gland. Bar: 20 µm.

We have previously shown using RT-PCR experiments on RNA prepared from salivary gland dissected lobes that PRS1 was expressed at higher levels in the lateral distal lobe than in the lateral proximal or in the medial lobes [15]. Immunolocalization allowed PRS1 detection in infected and non-infected cells of the distal portion of the lateral lobes with PRS1 immunoreactivity located at the basal side of the salivary gland cells (Fig. 4). Less or no immunoreactivity was observed in the proximal lateral lobe while interpretation of immunolocalization was hampered in the medial lobe due to a high level of autofluorescence. In the distal lateral lobes, no immunoreactivity was detected in the central parts of the glands corresponding to the secretory cavities and ducts, in agreement with the notion that PRS1 is an intra-cellular, cytoplasmic protein (Fig. 4C and Video S1). Notably, while PRS1 labeling in cytoplasm was homogeneous in non-infected and some infected cells, it displayed a granular aspect in highly infected cells suggesting a relocalization of the protein to vesicle-like structures (Fig. 4 A, B, C, E). These granules did not colocalize with GFP labeled sporozoites, indicating that PRS1 relocalization likely reflects an intra-cellular response to invasion rather than a direct interaction with invading sporozoites (Fig. 4C).

Intriguingly, we observed that, in poorly infected glands, sporozoites were not homogeneously distributed in the whole gland but were concentrated in one or several foci of highly infected cells surrounded by uninfected regions (Fig. 4 D, G, I). In these foci, an increase in the density of the actin network and a modification of cell shape were often noticeable (Fig. 4F and Fig. S4), suggesting alterations in the cytoskeleton. As the glands were more densely infected, the foci were larger, sometimes covering only one side of the gland (Figure S4). Finally, glands that were completely infected were also observed. These observations suggested that invasion of the salivary glands is a progressive process beginning with primary foci of infection that can extend to the whole gland, rather than a general invasion process of each salivary gland cell.

Furthermore, a higher PRS1 immunoreactivity was detected in the foci corresponding to highly infected cells compared to surrounding uninfected regions (Fig. 4. E, H, I). As PRS1 expression increased in infected cells, a prediction was that the levels of PRS1 expression might directly reflect the intensity of gland infection. To explore this hypothesis, we quantified *PRS1* and *P. berghei* Circumsporozoite (Cs) protein mRNA in the salivary glands of infected *An. gambiae*. In these experiments, Cs mRNA was used to quantify the density of invasion. Strikingly we observed a strong correlation (correlation coefficient R2 = 0.7, p<0.001) between the expression levels for the two mRNAs showing a clear relationship between PRS1 expression and salivary gland invasion (Figure S5). Collectively, our results support the idea that sporozoite invasion of salivary gland cells occurs sequentially and induces intra-cellular modifications that include an increase in *PRS1* expression and a relocalization of the corresponding protein into vesicle-like structures.

### 5) PRS1 has a facilitator role in *Plasmodium* infection

The expression profile and the overexpression and relocalization of PRS1 in both the midgut and the salivary glands in response to *Plasmodium* invasion strongly suggest that PRS1 is involved in *Anopheles*/*Plasmodium* interactions. To determine the role of PRS1 on *Plasmodium* development in the mosquito, we investigated the effect of *PRS1* silencing by RNAi. Two different protocols were used to differentiate the potential effects in the midgut and the salivary gland cells. First, to detect *PRS1* knockdown (KD) effects on midgut invasion by ookinetes, *PRS1* dsRNA and control dsRNA were injected three days before the infective BM. Second, to detect specific effects on sporozoite development and salivary gland invasion, independently from the effects on oocyst development, *PRS1* and *GFP* dsRNAs were injected into the hemocoel 7 to 10 days after the infective BM, at which time oocysts are already formed and sporozoites are in the process of salivary gland invasion. These protocols were used for assessing *PRS1* silencing effects in both the *P. berghei* and *P. falciparum* systems.

**Figure 5:**
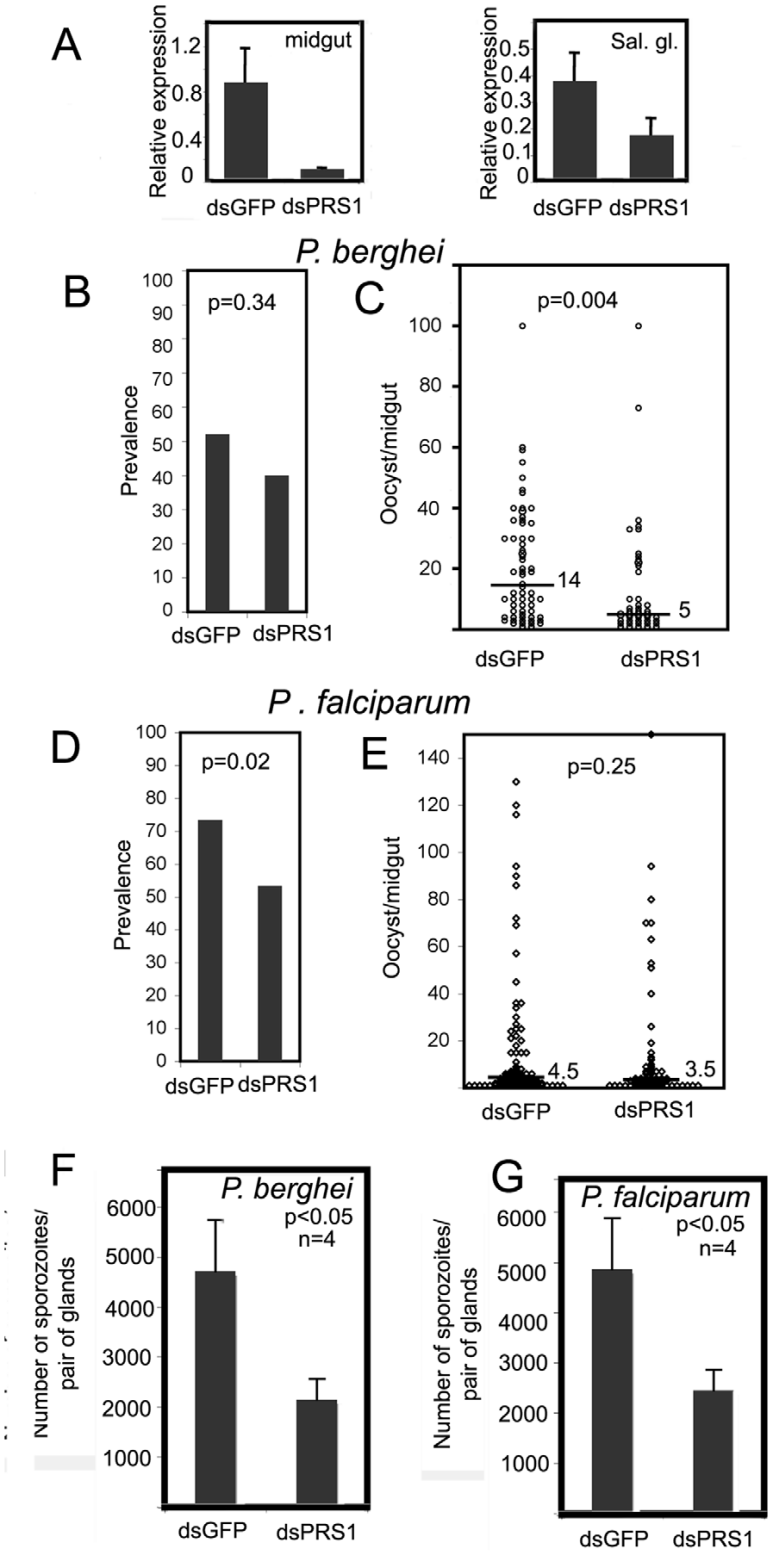
*PRS1* affects *An. gambiae* infection with *Plasmodium*. A. Effects of dsRNA injection on *PRS1* expression in the midgut and the salivary glands. Expression of *PRS1* was analyzed by qRT-PCR in the midgut and the salivary glands three days after injection of ds*PRS*1 or control ds*GFP*. For PRS1 silencing in salivary glands, three fold higher amounts of control or specific dsRNA were injected. B–E. Oocyst prevalences and oocyst intensities in *P. berghei* (B, C) or *P. falciparum* (D, E) infected mosquitoes after injection of *GFP* dsRNAs (controls) or *PRS1* dsRNAs. For each infecting parasite, data from four independent experiments were pooled and analyzed for prevalence values (B, D) or distribution of oocyst intensities in positive midguts (C, E). In C and E, the black bar represents the cumulated median intensity value. Statistics were computed on each individual replicate (also see Sup. Table S3) and the p values were combined using the meta-analytical approach of Fisher as described in Experimental Procedures. Injection of PRS1 dsRNA significantly decreases prevalence in *P. falciparum* infections and oocyst intensities in *P. berghei* infections compared to injection of control dsRNA. F and G. Average parasite numbers in salivary glands after infection by *P. berghei* (F) or *P. falciparum* (G) of mosquitoes treated with *PRS1* and *GFP* dsRNA. Data were collected from four independent experiments. The standard errors are indicated. Statistically significant differences between samples were evaluated using the Mann-Whitney and Student's tests. The p-values reveal significant differences between ds*GFP* controls and ds*PRS1* KD.

QRT-PCR analysis of midgut and salivary gland RNA indicated a significant *PRS1* silencing in both tissues (Fig. 5A). Nevertheless, gene silencing in the salivary gland was less efficient despite the use of larger amounts of dsRNA, in agreement with previous observations [21].

The effects of gene silencing on oocyst development in the midgut were tested in four biological experiments, with each parasite. Since differences between replicates were observed within treatments, the data were not pooled for statistical analyses. The replicates were instead analyzed independently and resulting p values were combined by meta-analysis (cf Experimental Procedures). Infection prevalence, i.e. the fraction of mosquitoes carrying at least one oocyst, and infection intensity, i.e; the number of oocysts per mosquito determined only in the subset of mosquitoes with >0 oocyst, were separately tested. Injection of *PRS1* dsRNA before infecting mosquitoes by *P.berghei* significantly decreases the infection intensity (p = 0.004) but the effect on prevalence was not significant (p = 0.18) (Fig. 2B, C). In contrast, *PRS1* knockdown was found to affect *P. falciparum* infection more at the level of the prevalence of infection (p = 0.02) than at the level of oocyst intensity (p = 0.25) (Fig. 2D and E). The details of the analyses are given in Table S2. The reasons while PRS1 silencing differentially affects prevalence or intensity for *P.berghei* or *P.falciparum* are currently unknown and might be linked to *Plasmodium* specific biological effects. Indeed, *P. falciparum* was observed to usually display a weaker infection against its natural vector *An. gambiae* compared to *P. berghei*, probably reflecting co-evolution between this mosquito and the human malaria parasite. This is in accordance with field data [20], [22] showing that *An. gambiae* resistance against the human malaria parasite is a relatively frequent trait. In addition, in our experimental conditions, *P. berghei* infections lead to a number of developing oocysts higher than *P. falciparum* infections, with a significant difference (Wilcoxon test W = 7993.5, p-value  = 0.004727). Nevertheless, despite this parasite species-associated difference, the decrease in the efficiency of infection in response to *PRS1* silencing showed that *PRS1* is an agonist of oocyst development in both *Plasmodium* systems.

Finally, we investigated the effects of *PRS1* silencing on *Plasmodium* sporozoite invasion of salivary glands. *PRS1* and *GFP* dsRNAs were injected into the mosquito hemocoel just before sporozoites start invading salivary glands. Consistent with the reduction in PRS1 expression, we observed a significant, 2-fold, decrease in the number of sporozoites in salivary glands at day 16 (*P.berghei*, Fig. 5F) and day 11 (*P. falciparum*, Fig. 5G) after infection. Therefore, PRS1 appears to be necessary for efficient salivary gland colonization by both *P. berghei* and *P. falciparum*.

Jointly these results show that PRS1 is an agonist of *Plasmodium* development at the two steps where the parasite has to invade mosquito epithelial barriers, irrespective of the *Plasmodium* system under study.

## Discussion

We previously identified several *Anopheles gambiae* genes that were differentially expressed in infected salivary glands. Here, we focused our study on the *PRS1* gene, a member of a novel superfamily essentially found in arthropods and characterized by the DM9 protein domain. Most importantly, our data identify PRS1 as an agonist of *P. falciparum* development acting at the two epithelial barriers crossed by mobile forms of the parasite, i.e. ookinetes and sporozoites. PRS1 function, in both epithelia, appears to be conserved in the compatible but non-natural *P. berghei/An. gambiae* system, suggesting that PRS1 is involved in biological processes exploited, or selected by a broader range of pathogens or parasites, which *An.gambiae* adult or larvae can encounter.

While *PRS1* expression in the salivary glands was previously established, our study shows that *PRS1* is also expressed in the midgut where it is upregulated after blood-meal. In addition, *PRS1* expression at the mRNA level is significantly higher after an infecting compared to a non-infecting blood-meal by 24–30 h, coincident with the peaks of *P. berghei* and P. *falciparum* ookinete invasion. To further link the induction of *PRS1* expression at 24–30 h with midgut epithelium invasion by ookinetes, immunocytochemical detection of PRS1 expressing cells was performed. PRS1 overexpression was mainly observed in groups of cells associated with ookinetes and protruding towards the midgut lumen. According to the “time bomb” theory of *P. berghei* ookinete invasion [6], ookinetes traverse midgut epithelial cells, often serially, and trigger apoptosis and extrusion of the invaded cells from the epithelial layer. Interestingly, in these cells, PRS1 induction is followed by relocalization of the protein at the cell periphery, indicating that PRS1 might participate in cell responses to damage or apoptosis. PRS1 overexpression was also observed in the proximity of basally located ookinetes, without any sign of apoptosis in the surrounding cells, suggesting that the apoptotic cells have already been expelled. Alternatively, ookinete invasion may not systematically lead to an apoptotic process, as also suggested by Shiao et al. [9].

While each midgut cell is in mean the target for less than one ookinete, salivary gland cells are invaded by numerous sporozoites. This probably leads to an intensive cellular repair process, the mechanism of which is currently unknown. Among the changes occurring in invaded salivary gland cells, Sterling et al [5] have previously noticed the accumulation of small membrane-limited vacuoles. Interestingly, PRS1, as well as the Serpin 6, another protein whose expression is induced during salivary gland invasion by sporozoites, were found to concentrate into vesicle-like structures. It may be hypothesized that formation of these vesicles is linked to the intense protein and/or membrane synthesis required to repair cell damages. Whether there is one or more types of vesicule-like structures and whether proteins with different functions, agonist for PRS1 and antagonist for Serpin 6, are colocalized in the same vesicles remain to be established.

Strikingly, we observed that, in the distal lateral lobes of the salivary glands, sporozoites were often heterogeneously distributed suggesting that there may be preferential routes for salivary gland invasion. It was proposed that hemolymph flow through the dorsal vessel may explain preferential routing of sporozoites to salivary glands [23]. This might also explain why some regions are more accessible to sporozoite invasion. Alternatively, partial degradation of the basal matrix by earlier invading parasites, or a localized shutdown of the epithelial immune response, might facilitate further penetration for the trailing sporozoites. Nevertheless, the observed “quantum-like” invasion of salivary glands, where a cell is either not invaded or highly invaded, is likely to explain the observed pronounced correlation between the level of PRS1 mRNA induction and the number of sporozoites inside the gland.

Our immunolocalization results reveal a link between epithelial cell invasion and PRS1 overexpression, however the mechanism of PRS1 induction remains to be established. Epithelial invasion by pathogens, particularly in the midgut, was often associated with the induction of the immune system [24], [25]. Recent articles have demonstrated the major roles of Toll and Imd signaling pathways, mediated by Rel1 and Rel2 respectively, in triggering immune responses to *Plasmodium* in the midgut [25], [26], [27]. Furthermore, RNA interference to deplete the negative regulators of these pathways, Cactus and Caspar, combined to high throughput gene expression analyses highlighted 588 and 116 genes, respectively, regulated by Toll and Imd pathways [28]. However, this list is probably not complete since some genes, like PRS1, were not probed in these analyses. Therefore, it would be worth to investigate whether the induction of PRS1 could be linked somehow to the induction of the immune system. A double knockdown affecting both PRS1 together with one of the two immune pathways, Toll or Imd, by targeting their respective downstream transcription factor Rel1 or Rel2, could help to address this issue, in the midgut but also in the salivary gland.

Whatever the mechanism controlling PRS1 induction, our work shows that *PRS1* silencing similarly affects *Plasmodium* infection in the two mosquito epithelia crossed by the parasite, reducing the number of oocysts that develop in the midgut and the number of sporozoites that colonize the gland. Interestingly, the phenotypes obtained after PRS1 gene silencing were largely similar with both *P. falciparum* and *P. berghei* species. RNAi silencing assays were previously used to test a number of potential effectors of the immune response against *Plasmodium* infection. Several highly potent immune genes were shown to exert agonist or antagonist *Plasmodium* species-specific effects, suggesting adaptation of the innate immune response during co-evolution between parasite and its natural vector. In contrast, other aspects of mosquito/parasite interactions were found to be evolutionarily more conserved. For instance, silencing the WASP gene, a regulator of the cytoskeleton dynamics increased the number of developing oocysts by a two-fold factor using either *P. berghei* or *P. falciparum* [10]. Similarly, PRS1 might be involved in a cellular activity stimulated by invasion, such as cytoskeleton reorganization or membrane synthesis that could *in fine* modulate *Plasmodium* survival. We can hypothesize that such an activity would escape the selective pressure imposed, in the field, by at least *P. falciparum.* This would make sense with the conservation of WASP and PRS1 functions towards both parasite species.

Future studies will be required to determine the molecular mechanisms underlying PRS1 functions as a general agonist of *Plasmodium* development. At the structural level, PRS1 is only composed of two DM9 motifs. To date, the functions of these motifs are still unknown. However, three *Drosophila* proteins carrying these motifs (CG10527, CG3884 and CG13321) have been shown to interact with various proteins involved in transcriptional regulation, intra-cellular trafficking, cytoskeleton rearrangement and immune responses [29], suggesting that they could be involved in regulatory protein-protein networks. The same three proteins were detected in the *Drosophila* phagosome [30], a localization compatible with a function in vesicular trafficking or immune response. A fourth *Drosophila* DM9-containing protein (CG16775) was also described to be strongly up-regulated after oral infection of *Drosophila* larvae by an entomopathogenic *Pseudomonas* species [31]. Altogether, these data suggest that DM9-containing proteins are involved in regulatory interactions during local immune responses. PRS1 relocalization at pericytoplasmic sites in the midgut or in vesicle-like structures in the salivary glands is compatible with a link to intracellular trafficking. It will be of further interest to determine whether other *Anopheles* DM9 motif proteins may have a role during ookinete invasion, which could be associated with cell damage, re-organization of the cytoskeleton or in immune defenses against pathogens.

In conclusion, previous studies have fostered the concept of potentially universal *Plasmodium* agonists at least for parasite development in the midgut. Our results, which showed that PRS1 is an agonist of the *Plasmodium* developmental cycle both in the midgut and in the salivary glands, for the human and the rodent parasites, strengthen this notion of fundamental mechanisms involved in the mosquito epithelial invasion by pathogens independently of parasite/vector coadaptations.

## Materials and Methods

### Mosquito rearing and infection

Mosquito (*An. gambiae* Yaounde strain) rearing and infection by the rodent parasite *P. berghei* were conducted as previously described [15]. Two *P. berghei* strains were used: the PbFluspo strain [32] for expression studies, gene inactivation and detection of sporozoites and the conF strain [33] for detection of earlier *Plasmodium* forms during midgut invasion. For expression studies, mosquito infection with *P. falciparum* was initially performed in Senegal, using blood of gametocyte carrier volunteers, as described [34]. Salivary gland infection by *P. falciparum* was confirmed by PCR detection of aldolase mRNA (*PF14_0425*). Other studies with *P. falciparum*, especially RNA silencing experiments, used infections with gametocytes produced *in vitro* (as described further).

### *In vitro* production of *P. falciparum* gametocytes and mosquito infection procedure

The *P. falciparum* NF54 isolate was cultured using the tipper table system [35]. Briefly, parasite culture was initiated with fresh human red blood cells (RBCs,7% final concentration) in RPMI 1640 medium, containing 25 mM HEPES and L-glutamine, 10% heat-inactivated human serum and 0.2% Sodium Bicarbonate, under a constant gas supply. Fourteen days later, gametocyte maturity was verified by testing microgamete exflagellation, and parasitemia quantified on Giemsa-stained slides. After centrifugation at 2000 rpm, gametocytes were resuspended in an equal volume of AB human serum and added to a mixture of fresh RBCs and AB human serum. Mosquitoes were allowed to feed on that mixture for 15 minutes, using a membrane feeder, previously warmed to 37°C. Only fully fed females were kept for further analysis.

### Real-time quantitative RT-PCR

Mosquito tissues were dissected in RNAlater (Ambion; 70% in PBS) and frozen in 100% RNAlater at –80°C until utilized. Total RNA was isolated using Tri reagent (Sigma, USA). Real-time PCR was performed on cDNA preparations using the SYBR green detection system (Applied Biosystems, Warrington, UK) with the ABI Prism 7900 sequence detector (Applied Biosystems), as previously described [15]. The *An. gambiae* RPS7 ribosomal gene was used as an internal control to normalize the amounts of RNA between the various samples. In each experiment, a mixture of RNAS was used for drawing standard curves for the quantifications of RPS7 RNA and the RNA under study. This mixture of RNA was defined to have an expression level of 1 under its non diluted form. Sequences of the primers are listed in Table S3.

### Recombinant protein and antibody production

The cDNA clone BX037582, obtained from an *An. gambiae* full-length cDNA library [18], was resequenced and used to generate a PRS1-GST fusion protein. Briefly, a 480 bp PCR fragment was obtained using oligos PRSrec5′ and PRS1rec3′ (Table S3) and inserted into a PGEX6P1 vector (Amersham biosciences). The 388 AA-long fusion protein carrying GST on the N-terminal end was purified on a glutathione affinity column (Amersham), and elution was performed by cleavage with the PreScission protease (Amersham) according to the manufacturer's instructions. Cleavage using the PreScission protease releases an 18 KDa protein with an 18 AA N-terminal extension. This recombinant protein was used to raise rabbit polyclonal antibodies (SE 6119, Eurogentec).

### Western blotting

Midguts and salivary glands were dissected in PBS, placed in ice-cold 50 mM Tris at pH 8, 100 mM NaCl, 5 mM EDTA, and homogenized. Samples were separated on 15% SDS-PAGE or 15% NuPAGE Novex (Invitrogen) precast gels, followed by electrotransfer to a Nitrocellulose Protran membrane (Whatman, Schleicher and Schuell) and incubation with antibodies against PRS1 (1∶2500) or Actin (clone 20–33, 1∶500, Sigma). Primary antibodies were detected using goat anti-rabbit peroxidase-labeled antibodies (A0545, 1∶75000, Sigma) and visualized using the ECL Plus Western Blotting Detection System (Amersham). Blots were stripped with Restore Western Blot stripping Buffer (Thermoscientific).

### Immunostaining for confocal microscopy

Salivary glands were dissected in PBS and fixed for 15 min in paraformaldehyde (PFA; 4% in PBS). Dissected midguts were treated as described [36], except that a 4% PFA solution in PBS was used for fixation. Fixed tissues were incubated with anti-PRS1 antibodies (1∶200) (2 h at RT for salivary glands or O/N at 4°C for midguts), followed by secondary alexafluor 546-labeled goat anti-rabbit antibodies (1∶500, Invitrogen). Actin labeling was performed by incubating salivary glands for 20 min with alexafluor phalloidin 633 (Invitrogen) in PBS plus 1% BSA. Samples were mounted in Prolong Gold Antifade reagent plus DAPI (Invitrogen) and analyzed using a Leica SPE confocal microscope. Image analyses were performed using Image J. For measures of the mean fluorescence intensities on midgut sections, the specimen were treated in the same way in parallel experiments and the image acquisition parameters were maintained the same.

### RNAi gene-silencing assays

Double-stranded RNAs were synthesized from PCR-amplified fragments using the T7 Megascript RNAi Kit (Ambion) according to the manufacturer's protocol. Primer sequences are listed in Tab. S3. Ds*GFP* was used as a control for non-specific effects of dsRNA injection. A quantity of 500 ng of dsRNA was injected into the thorax of cold-anesthetized 1- to 3-day-old *An. gambiae* females using a nano-injector (Nanoject II; Drummond) with a glass capillary needle, according to established methodology [37]. Alternatively, 1000–1500 ng were injected into the thorax of 13- to 14-day-old females at 10 days post-bloodmeal (d. PBM) (*P. berghei*) or 6 d. PBM (*P. falciparum*). Mosquito midguts were dissected at 8–10 d. PBM, and oocysts were counted after staining the midgut with 0.2% mercurochrome (*P. falciparum* infections), or using fluorescence microscopy (*P. berghei* infections). For statistical analysis, replicates were analyzed independently using Chi Square for differences in prevalence and non parametric Mann-Whitney test and the p values from independent tests of significance were combined using the meta-analytical approach of Fischer [38] as described [27]. Sporozoites in pools of glands were counted by light (at 14 d. PBM for *P. falciparum*) or fluorescence (at 16 d. PBM for *P. berghei*) microscopy using a hemocytometer and differences in sporozoite numbers were tested using non parametric Mann-Whitney test.

### Phylogenetic analysis

Sequence alignments of DM9-proteins with CLUSTAL-W and phylogenetic analysis using Phylip neighbor-joining method on distances were carried out using the Mobyle portal of the Institut Pasteur (http://mobyle.pasteur.fr).

## Supporting Information

Table S1 List of the proteins containing DM9 motifs.
(0.21 MB DOC)

Table S2 Effects of PRS1 RNAi silencing on Plasmodium development Details of the prevalences and oocyst densities obtained for the four infections after injection of GFP dsRNA or PRS1 dsRNA using P. berghei or P. falciparum, as shown in Fig. 5.
(0.08 MB DOC)

Table S3 Primers used in the study
(0.03 MB DOC)

Figure S1 Alignment of DM9-containing proteins belonging to PRS1 subfamily. DM9 proteins whose genes are located on chromosome 2 in An. gambiae and their closest homologues in An. gambiae (Ag), An. darlingi, Culex quinquefasciatus (Cq), A. aegypti (Aa), Phlebotomus papatasi (Pp) and Nasonnia vitripennis (NaV) were aligned together with two DM9 proteins from Drosophila (Dm: D. melanogaster) and this alignment was used for the tree shown in Fig. 1. The complete names of the proteins and their accession numbers are given in Tab. S1. Identical or similar residues found in more than 60% of protein sequences are highlighted in red and blue, respectively. DM9 motifs are indicated by double-ended arrows
(4.57 MB TIF)

Figure S2 Phylogenetic tree showing the relationships between in DM9 proteins found in An. gambiae (Ag), An. darlingi (Ad), Culex quinquefasciatus (Cq), A. aegypti (Aa), Phlebotomus papatasi (Pp), Nasonnia vitripennis (NaV) and D. melanogaster (Dm). The tree is unrooted. Bootstrap values superior to 75 per cent are indicated. Scale bar represents 10% differences in protein sequences.
(0.97 MB PDF)

Figure S3 Immunoblots of midgut and salivary gland extracts using anti-PRS1 antibodies. Molecular masses of the markers are indicated in kDa. A. Immunoblot of midgut and salivary gland extracts from non-infected An. gambiae. B. Immunoblot of An. gambiae midgut sheets before (time 0) or at various times (24 h, 48 h, 72 h) after an infected (+) or a non-infected (−) BM.
(3.76 MB TIF)

Figure S4 Phalloidin labeling of the actin network in salivary glands. A: Focal sections from upper to lower sections showing merge labeling for actin (red), GFP (green) and DAPI (blue). The number in the lower left corner indicates the number of the section. Bar: 20 µm. B: Z stack projection of all the sections. The white line indicates the direction of the plane used in C for 3-D reconstruction. C: 3-D reconstruction of a cross-section of the gland according to the direction defined by the white line in B. Note that sporozoites are localized in close proximity to the actin network.
(3.27 MB TIF)

Figure S5 Correlation between PRS1 and Cs expression in salivary glands; mRNAs for PRS1 and CS were quantified by qRT-PCR in different preparations of salivary glands after invasion by P. berghei. CS expression is used as a marker of the number of sporozoites inside the glands. A correlation between both sets of data is demonstrated by the Pearson correlation coefficient (R = 0.84; R2 = 0.7, p<0.001).
(2.00 MB TIF)

Video S1 3D-reconstruction of an infected salivary gland. 3D reconstruction after confocal microscopy of a salivary gland invaded by sporozoites (green) and stained for PRS1 (red) and DAPI (blue) Note the granular pattern of PRS1 labeling at the periphery of the gland contrasting with a more internal localization of most sporozoites found in bundles near the salivary duct.
(6.41 MB MOV)
