# Supplementary material for: Anopheles Gambiae PRS1 Modulates Plasmodium Development at Both Midgut and Salivary Gland Steps
Source: PLoS One. 2010 Jul 12;5(7):e11538. doi: 10.1371/journal.pone.0011538 (PMC2902509; doi:10.1371/journal.pone.0011538)
Supplement: Table S1 — List of the proteins containing DM9 motifs. (0.21 MB DOC) [file pone.0011538.s001.doc]

**DM9 motif-containing proteins detected by blast-p analysis on non-redundant protein sequences database at NCBi ( e-value <1)**

**Sequences used for multiple alignment and tree**

| Complete name | Short name^a^ | Species | Sequence^b^ |
| --- | --- | --- | --- |
| gi|157109281|ref|XP_001650602.1| | Aa6103 | Aedes aegypti | MSYITGKIKCPLACNYANWQTSFVNTGNSFGGCWQHCNIDGPFPTNMVRAGVDADGSVIFAGRAFHEGEMIPAKVIPSKNACYICYGG  EEIMKEDFEVLRQGDFVWEFAANGVVPDGAVKMGATVDGEPLYMGRALHCGTQTPGKVHSSHGCLYIPFEGAEISHAEYEVLCLK |
| gi|157128533|ref|XP_001661472.1| | Aa6398 | Aedes aegypti | MNAGYNWMPWSAHQGIPPAGVYAGNDQDGSPIYIGRAFHEGDQLPAKVIPSKQAAYVSHNGVEIYKSHFEVLSGQGFTWVHSGNGHV  PTGAVLCGNTVSGEPLYIGRTHHEGSLTPGKIHRSHGCLYIPFGGAEQSFLNYEVLVGQQKST |
| gi|157109287|ref|XP_001650605.1| | AaPRS1 | Aedes aegypti | MATKWVWTNAHGPYPPNMVSGGQDSDGALLYVGRANHAGDVLPAKVIPQKNAAYIAYGGEEVLVENFEVLCQKELIWDSATGGNIPP  DAVVGGNTADGEPLYIGRAYHEGSQTIGKVQRSHGCCYIPYGGAEVSVPTYDVLCER |
| gi|208657577|gb|ACI30085.1| | AdPRS1 | Anopheles darlingi | MATNWVPISVHGPHPPHMVPGGTDSDGCQIFVGRAHHAGDLLPAKVIPDKNAAYVPYGGQETFVDQVEVLVHKQLIWDVASSGQVPLG  AIIGGHTSDGETLYVGRTYHEGSHTVGKVQCSHNCIYIPYGGAEVSLPTYEVLCER |
| gi|157128531|ref|XP_001661471.1| | Ae28531 | Aedes aegypti | PAHTWVQWSADSAVQPPKAVKAGADHDGSPIYVGRASHYGDMLPAKVIPLRRVAYVTHDGLELSKPRFEVLCGLGFTWVPCENGNLPK  GAVLCGKTAYGEQLYIGRAHHNGSVTPGKIIRSHGCLYIGFDGVELAHPKYEVLVD |
| gi|157115555|ref|XP_001658262.1| | Ae9604 | Aedes aegypti | AALVSSNAACWIPAANGEIPPNAVVGGSDGEDMYIARAQHEGAIIPGKLLASHGAAYVAWGGAENPKTEYEVLCDGNGTFVPTSGGEIPP  NAIPAGESEDGEPLFIGRVAHEGTMTVGKVQQSHGVCYIPYGGQEMAFADYEIYVSQ |
| gi|157115553|ref|XP_001658261.1| | Ae9605 | Aedes aegypti | MVHYASLYRDYIPFHADRPYKYHNRRFLERWDKLNGMQWVKASNGEIPPNAVIAGHEGNQTLYVGRAEVNNSIAPGSVNPQKRACFCP  WGGKNHKRPTYEVLCTPGQFVEVDSWNTLVLGTPGGISEQGEPLYIGRNVQNSELISGKIQRSYFVCYIPYKTKEVERPVFGSQIFIKSTS |
| gi|157115551|ref|XP_001658260.1| | Ae9606 | Aedes aegypti | lFVANVPMRLDSSYWLPNFKSDIPEHATVGGGTPNKSLYIGRAKHRGSLTPGSVDPETWQCHIAWGSDEHRKTYFEYLCRCSGRFVKSQG  NHLPIGAIRGGYSEYGEPLFIGRVKMKEGYIVGKVQPSHAVCYIPYRGKEIAYKKYEILVQAD |
| gi|158295345| AGAP006103-PA | Ag6103 | Anopheles gambiae | MITSCWQHCNVNGPFPPNMVRAGVDSDGEVIYVGRAFHEGDMVPAKVIPTKNVAFVCHGGEEVLKEDFEVLRYGAFVWEYSSNGSVPET  AMRIGQTMDGEPLYMGRAIYSGSQTPGKVHPSHGCCYLPFDGAEVSVTDYEVLCIR |
| gi|158295808| AGAP006398-PA | Ag6398 | Anopheles gambiae | MNTGFNWIPWTSHQGIPPAAVYGGNDQDGSPIYIGRAYHEGDQLPAKVIPSKQAAYVSHNGMEIFKTHFEVLTGTGFTWVSSGNGHVPA  NAVLAGNTTTGEQLYIGRTHHEGSLTPGKIHRSHGCLYIPFGGAEQSFLSYEVLVGQQRSN |
| gi|158298459| AGAP009604-PA | Ag9604 | Anopheles gambiae | AAALSAGGAACWVAAANGEIPPNAVVGGSDGEDMYIGRAQHEGGIIPGKVVASHGVCYIAWGGAENPKAEYEVLCDFGGEFVPASGSD  IPPTALPAGESEDGEPLFIGRVTHEGTVTVGKVQPSHGVCYIPYGGQELAFAEYEIFVSP |
| gi|118790503| AGAP009605-PA | Ag9605 | Anopheles gambiae | LHSMKWVPYQDSGPLPPSAVECGTSKRTKLYLGRAEHAGSVTPGFINPAKKVCYIPWGGKAHEKKVCEILCTAGEFVPCTETNVLLRATP  AGVSEQGEPLYIGRVAVDGQLVCGKVQRSHSVCYIPYNRKEEPHVNFEVFIKSQQ |
| gi|158298461| AGAP009606-PA | Ag9606 | Anopheles gambiae | LDNARWVQAAEGLVPPDAVVGGYEGEVTFIGRAKHRGSIVPGRIVPSKKACCVVWGGEEHTKSDYQVLCGYEGHFVHVGGGYIPNGALR  GGVSEHGKPLYIGLVRLGSTTVVGKVQPEHSCCYIAVGGVEKAFREYDVYVTTAGYNQPVPGR |
| gi|158295339| AGAP006102-PA | AgPRS1 | Anopheles gambiae | MATTWIPTSVHGPYPPHMVPGGVDSDGAQIFVGRAHHAGDLLPAKVIPDKTAAYVAYGGQETLVEHVEVLVHKQLIWDTASAGQVPLGA  VVGGHTSDGEILYVGRAYHEGSQTIGKVQCSHNCIYIPYGGAEVSVPTYEVLCER |
| gi|104532027|gb|ABF72903.1| | BA2027 | Belgica antarctica | MLEIFIGGWGNKKSVIRRNRSKPDVVEVETPNILSAGEFKGFWVRWDNGNITVGHEGEAASFLSYQNPNPFPINFIGLCTGWGASGSWVLDT  PQGSASRWLPQGAQGGASRWLPQGAQGGNAVWVGASGSNIPSGAFVGGHDNGEGLVVGRAHHEGALIPGKVVPSHGVCYVAWGRR |
| gi|170036503|ref|XP_001846103.1| | Cq36503 | Culex quinquefasciatus | PPEPGTVGHYWRSTEEFSVPANATIGGSNEHGPLYIGHASHRGSCTPGQISRVTNRCHIAWGGSEHRKPAFEFLCNCRGRFVASQEGQVPV  GAILGGWSEYEGEPLFVGRVQVKGHWLVGKLQPSHKVCYIPINGKEVAHKHYEIFVQDGI |
| gi|170036505|ref|XP_001846104.1| | Cq36505 | Culex quinquefasciatus | QRRDHNSGLRWIWASDGEVPPNAVKTGEGCYLGRAFHVGSVTPGRVDPEKKACCIPWGGDEHLKKVYEVLCTEGEFVRVTPDSTEGLLRA  TTAGISEQGEPLFIGRVWCDGGWVSGKVQRSHGVCYIGYRGKEMAYPEYEVFVGV |
| gi|170052249|ref|XP_001862136.1 | Cq6103 | Culex quinquefasciatus | MTGGCWQHCNVGGPFPANMVRAGVDADGSVIYAGRAFHEGEMIPAKVIPSKNEAYICFGGEEILKEDFEVLRAGDFVWEFAANGVVPEGA  IKMGATVDGEPLYMGRALHCGTQTPGKVHSSHGCLYIPFDGAEISLPEYEVLCIK |
| gi|170063139|ref|XP_001866972.1| | Cq63139 | Culex quinquefasciatus | MKRCAVLKYLTWLPRKNYQDPPEGTVLAGYDSDQSPIYVGRVMYEGNQLPAKVIPRKQLCHSQYNGEEIEMISYEALCHGNVEWVRFRGT  IPANAVVCGRLISGEVVHIGRGSHLGSLTPGRVLSGEKVLYIPFGWNEIRITDFEILIDN |
| gi|170063143|ref|XP_001866974.1| | Cq63143 | Culex quinquefasciatus | EVYNWVPWSADAGVHPANAVRAGTDTDGSEIYVGRAEHYGEMLPVKFLPKRRVAYVSQGGIEFFKAHFEVLTGLSFTWVPAVNGEIP  KGAVFVGKTTHGEPMYIGRGHLDGSVTPGKVLKSQGCLYVPFGGFEQATSKYEVLVDG |
| gi|170063137|ref|XP_001866971.1| | Cq6398 | Culex quinquefasciatus | MNAGYNWMPWSAHQGNPPAGVYAGNDQDGSPIFVGRAFHEGDQLPAKVLPSKQAAYVSHNGCEIFKSHFEVLSGTGFTWVHSSNGHVP  AGAVLCGNTTTGEPLYIGRAHHEGSLTPGKIHRSHGCLYIPFGGAEQSILHYEVLVGQQRSN |
| gi|170036501|ref|XP_001846102.1| | Cq9604 | Culex quinquefasciatus | SSGGAACWVPAANGEVPPNAVVGGADGEDMYIARSQHEGAIIPGKLVASHGCAYVAWGGVENPKQEYEVLCDGNGTFVSTSGGEIPP  NAIPAGESEDGEPLFIGRVNHEGTVTVGKVQQSHGVCYIPYGGQELAFADYEIYVSQ |
| gi|170036507|ref|XP_001846105.1| | Cq9605 | Culex quinquefasciatus | MVQYASLYDDQYRYRAERPYNHDKWKFLRRWDKLNGMTWIEASKGAVPPGAVVAGHQDGHTLYVGRAECMSSVAIGVVNPHRKA  CYVPWGGKSHKRETYEVLCTPGQFVPIDCCTTLLKGTPGGISEQGEPLYIGRTSHQGALIGGKIQRSYFFCYLPYKNREVERLVFESEIYIKSS |
| gi|170052252|ref|XP_001862137.1| | CqPRS1 | Culex quinquefasciatus | MATKWVWTNSAGPFPPNMVQGGQDSDGCGIFVGRANHNGDLLPAKVLPQKNAAYVAYGGEEVLVENFEVLCRKELVWEHATGGSVP  QEAVIGGNTGDGEILYVGRAYHEGSQTVGKVQRTHGCIYIPYGGAEVSLPSYEVLCER |
| gi|20130199| CG10527 | Dm10527 | Drosophila melanogaster | PTGSGPGCWVPAANGEVPPNALEGGFDSSEQLYIARARHEGDLIPGKLHPSHGVTYVAWGGGEHGHAEYEVLCAGGGQWLPVDAGNIPP  NALPAGETAEGEPLFIGRATHDGTITVGKVQPSHGCCYIPYGGEELAYKEFEIYVTN |
| gi|19922514| CG10916 | Dm10916 | Drosophila melanogaster | DSLPDAHLPPEGAVQCGTNEDGLPTYVARGYYHDDLLPAPYVPEKKAAFGSHSCSARTLTDDVEILVLNDCDYKWVPGQHGTYPRDALN  TGYSELGEVTYTGRGLYQGILRLGKVHPSHKVMYIPHHGQEVSVNTYEVLVVTPRDQADR |
| gi|19922144| CG13321 | Dm13321 | Drosophila melanogaster | MGDYTWISTNVYGSLPPGAILAGHDSDQDPIFVGRAYHNGEMLPAKVVPGKQQAYVPWGGQEISKHDFEVLVGDHFSWIPSSGGSVPPHA  IQVGQTGEGEPLYVGRGYFQGSLTPGKVHPSHQCLYIPYGGQEHRLEAYEVLVQPETWIA |
| gi|221512778| CG16775 | Dm16775 | Drosophila melanogaster | MFSAKSSAIVAVVLVQMVAQIHGGVYSYEDKWVYLDKTLDLPEEAILGGVDPDGYYTYVGRVTYSSNILPARVVPELGKATYNTDTLGN  QATTYEVLVSNATVGYHWIRSFDGFREKNAVSVGTNALSERVFICRVRCDESIFIGTLYLS |
| gi|24650270| CG31086 | Dm31086 | Drosophila melanogaster | MDGHSWLHFSNGAIPQAAVVAGHDSDGDTIFIGRAFYCNDMLPAKIIPNKGKAYVAYANQEVELENYEVLSGFNYEWLPAENGEVPPGAV  KVGQNVDGETLYAGRGYHAGSLTVGKVHPSHGCLYIPYDSEEVKIFAYEVLSRRLEAR |
| gi|18859785| CG32633 | Dm32633 | Drosophila melanogaster | MADHRWMHFSNGSVPPNAVVAGHDSDGDTIYVGRAFFSNDMLPAKVIPNKGKAYVAYAREEHELENYEVLSGYNYEWLSAENGEVPPG  AVKVGRNVDGEYLYAGRGYHAGSLTMGKVHPSHGCLYIPYDSDEVKIFAYEVLCQPERW |
| gi|24653247| CG3884, A | Dm3884-A | Drosophila melanogaster | MAYKWVQSSAYSSLPEEAVVGGNDEDGAMIYVGRAEHEGDMLVCKVVPSKQLGFISQRGEALPKDIFEVLCGQNLVWIKCYDHVIPENAV  LCGRTSLDQPVYIGRGHYEGHLIIGKISSVHRALFIAFRGAERRLDSYEILVEE |
| gi|19922142| CG3884, B | Dm3884-B | Drosophila melanogaster | MDNTWVHSSPYSPLPPYAVIGGHDSDRTPIYVGRSFHEGENLPAKVVPSKGCAYVAYGGAEHTKTHYEVLVGQGFAWVPSSSGGVPPNAV  RSGTTRTGEPLYVGRGHHAGSLTVGKVHPSHGCLYIPFGGQEVRINTYEVLIKQQHDVWV |
| gi|24666167| CG5506 | Dm5506 | Drosophila melanogaster | VWKAGNLSYVIPYNAVVGGFDPYGFTTYVGRVKYSNSILPARVVAETGTAYFNTETTSSKLLVYDILVAERDVNYVWVRSFDGFYEKGAV  AVGTTVKNERVFCCRAKTDGGILIGTLLLSSQKVCIIKHESLALRKFDKYEVLVAQPKGNGTYY |
| gi|157361527|gb|ABV44721.1| | Pp361527 | Phlebotomus papatasi | MTDFTWVPTSVYGAMPPNAVFAGTDSDSSPIYVGRTFHEGDQLPAKVIPSKQAAYVSHNGQEILKHHVEVLVGTGFTWIHSGNGHVPPNAV  RAGQTVHGHPLYVGRTHHEGSLTPGKIHAQHGCLYFPYGGAEQSSLQYEVLCGQPTSRW |
| gi|183584866|gb|ACC63901.1| | Cc584866 | Ceratitis capitata | MAEHHWVSTTIDSELPPFAVQAGHDSDGSPIFVGRAYHNGDMLVAKIVPNKGQAYVAWGGEEVNKHDIEVLTGHNYHWVPDANGAVPAG  AVACGQTSLGETLYVGRGYHADSLTPGKIHPSQGCLYIGFGGEEVTLTNYEVLVRNY |
| gi|189240245|ref|XP_001810808.1| | Tc40245 | Tribolium castaneum | KWDCADYYWRDYHETIPDDAIPAGTDSHGKPLYIGLAYVRGYELLPATILPSEKLARTTAYAKVFNTRDNVKILCSPYPEAFEWISIESRDL  HKYGKHNLIPGGSEVGENLYIGRVFRDNGVIVGKIFRHERQNRGIWFPLKNTHGNSLSYEILNYNCDSAIPKIDPRVNVE |
| gi|91078970|ref|XP_974395.1| | Tc78970 | Tribolium castaneum | APAMGSRGNFSNVCWVAARNGEVPPRAFAGGEDNGEPVYVARANFNGGLIPGKLVASHGTAYVPWGGQENAVPEYEVLCDFPGNWIACS  GGNVPPNAVTAGQSEEGEPLYVGRVVHDGSLTVGKVQPSHGVVYIPYGGTELGFQDYEILVQ |
| gi|91080431|ref|XP_968599.1| | Tc80431 | Tribolium castaneum | ADAIVGGRDSRGYPTYIGQAFVRCHGILIGQIYPGQKTITTSKEGIHVTDVYNRILCSGHKENFSWVPGNAATLHLTTINKHLVSGGTEWGKV  LNIGRVKYQGELIVGKVCSGTIGKAKLYFPYKGEEIESDTYEVLAYEDKPNEVESV |
| gi|91094473|ref|XP_970560.1| | Tc94473 | Tribolium castaneum | YYWVDTFTNGGVPSTALWGGEDIDGHQIYVGRAYFKNDWIPAKVIPGRRKAYVAYNGKEYTVDRFQVLCEQRFDWVKTTEDKIPEGAVEG  GRTVDGEPLYIGRVEHEGSHTVGKVHPSYKCCLIPFDGKELRFTEYEILILRP |
| gi|91094475|ref|XP_970632.1| | Tc94475 | Tribolium castaneum | fHNQAFRWVDSSIAYGSVPPTALQGGMDGDGHPIYVGRAYHEGDLIPAKVIPGKNAAYVSHNGQEHLVENFQVLCKQYFEWVQSHAGHL  PPGAVQGGHTSEGEPLYIGRAYHEGSQTIGKIHPSHGVCYIAYGGEEIACPEYETLVLRM |
| gi|57117776|gb|AAW34056.1| | Fh7776 | Fasciola hepatica | MHDGDMLPAKIVPRLGKAYVCHGGREHEYHSYEVLCDTKAPGTQKCYVWEHARGGHVPKYALLAGLSDSGDPIYVSRSEIDGERVVGKV  HSGHDCAYFPYGGREHQKSSYEVLVMKK |

a : abbreviated name used for the phylogenetic tree in Fig. 1 and Fig. S2

b : part of the protein sequence corresponding to two consecutive DM9 motifs and used for the phylogenetic tree in Fig.1, Fig. S2.

**Sequences not used for multiple sequence alignment and tree**

| Complete name | Species |
| --- | --- |
| gi|125776062|ref|XP_001359156.1| | Drosophila pseudoobscura pseudoobscura |
| gi|125809943|ref|XP_001361288.1| | Drosophila pseudoobscura pseudoobscura |
| gi|125811549|ref|XP_001361914.1| | Drosophila pseudoobscura pseudoobscura |
| gi|125979855|ref|XP_001353960.1| | Drosophila pseudoobscura pseudoobscura |
| gi|198457425|ref|XP_002138391.1| | Drosophila pseudoobscura pseudoobscura |
| gi|198457427|ref|XP_001360668.2| | Drosophila pseudoobscura pseudoobscura |
| gi|198457429|ref|XP_002138392.1| | Drosophila pseudoobscura pseudoobscura |
| gi|198466300|ref|XP_001353961.2| | Drosophila pseudoobscura pseudoobscura |
| gi|198466302|ref|XP_002135152.1| | Drosophila pseudoobscura pseudoobscura |
| gi|193713737|ref|XP_001944506.1| | Acyrthosiphon pisum |
| gi|193624888|ref|XP_001948032.1| | Acyrthosiphon pisum |
| gi|110755727|ref|XP_001122695.1| | Apis mellifera |
| gi|110768518|ref|XP_001122043.1| | Apis mellifera |
| gi|66508906|ref|XP_395998.2| | Apis mellifera |
| gi|66508910|ref|XP_623146.1| | Apis mellifera |
| gi|66508914|ref|XP_623207.1| | Apis mellifera |
| gi|110758543|ref|XP_001120831.1| | Apis mellifera |
| gi|148298754|ref|NP_001091815.1| | Bombyx mori |
| gi|194743798|ref|XP_001954387.1| | Drosophila ananassae |
| gi|194748068|ref|XP_001956471.1| | Drosophila ananassae |
| gi|194748070|ref|XP_001956472.1| | Drosophila ananassae |
| gi|194748072|ref|XP_001956473.1| | Drosophila ananassae |
| gi|194753904|ref|XP_001959245.1| | Drosophila ananassae |
| gi|194753906|ref|XP_001959246.1| | Drosophila ananassae |
| gi|194753908|ref|XP_001959247.1| | Drosophila ananassae |
| gi|194754539|ref|XP_001959552.1| | Drosophila ananassae |
| gi|194754541|ref|XP_001959553.1| | Drosophila ananassae |
| gi|194754543|ref|XP_001959554.1| | Drosophila ananassae |
| gi|194754765|ref|XP_001959665.1| | Drosophila ananassae |
| gi|194765729|ref|XP_001964979.1| | Drosophila ananassae |
| gi|194766227|ref|XP_001965226.1| | Drosophila ananassae |
| gi|194766229|ref|XP_001965227.1| | Drosophila ananassae |
| gi|194766961|ref|XP_001965587.1| | Drosophila ananassae |
| gi|194871667|ref|XP_001972883.1| | Drosophila erecta |
| gi|194871672|ref|XP_001972884.1| | Drosophila erecta |
| gi|194880991|ref|XP_001974632.1| | Drosophila erecta |
| gi|194880993|ref|XP_001974633.1| | Drosophila erecta |
| gi|194881782|ref|XP_001975000.1| | Drosophila erecta |
| gi|194882449|ref|XP_001975323.1| | Drosophila erecta |
| gi|194883478|ref|XP_001975828.1| | Drosophila erecta |
| gi|194883480|ref|XP_001975829.1| | Drosophila erecta |
| gi|194883482|ref|XP_001975830.1| | Drosophila erecta |
| gi|194895478|ref|XP_001978261.1| | Drosophila erecta |
| gi|194908041|ref|XP_001981692.1| | Drosophila erecta |
| gi|195027361|ref|XP_001986551.1| | Drosophila grimshawi |
| gi|195027956|ref|XP_001986848.1| | Drosophila grimshawi |
| gi|195027958|ref|XP_001986849.1| | Drosophila grimshawi |
| gi|195027960|ref|XP_001986850.1| | Drosophila grimshawi |
| gi|195028028|ref|XP_001986884.1| | Drosophila grimshawi |
| gi|195030520|ref|XP_001988116.1| | Drosophila grimshawi |
| gi|195030522|ref|XP_001988117.1| | Drosophila grimshawi |
| gi|195030524|ref|XP_001988118.1| | Drosophila grimshawi |
| gi|195030526|ref|XP_001988119.1| | Drosophila grimshawi |
| gi|195030528|ref|XP_001988120.1| | Drosophila grimshawi |
| gi|195038389|ref|XP_001990642.1| | Drosophila grimshawi |
| gi|195066917|ref|XP_001996857.1| | Drosophila grimshawi |
| gi|195067964|ref|XP_001996900.1| | Drosophila grimshawi |
| gi|195110381|ref|XP_001999760.1| | Drosophila mojavensis |
| gi|195113779|ref|XP_002001445.1| | Drosophila mojavensis |
| gi|195116241|ref|XP_002002664.1| | Drosophila mojavensis |
| gi|195116243|ref|XP_002002665.1| | Drosophila mojavensis |
| gi|195116259|ref|XP_002002673.1| | Drosophila mojavensis |
| gi|195120003|ref|XP_002004518.1| | Drosophila mojavensis |
| gi|195120005|ref|XP_002004519.1| | Drosophila mojavensis |
| gi|195120007|ref|XP_002004520.1| | Drosophila mojavensis |
| gi|195120381|ref|XP_002004707.1| | Drosophila mojavensis |
| gi|195122556|ref|XP_002005777.1| | Drosophila mojavensis |
| gi|195128965|ref|XP_002008929.1| | Drosophila mojavensis |
| gi|195150619|ref|XP_002016248.1| | Drosophila persimilis |
| gi|195150621|ref|XP_002016249.1| | Drosophila persimilis |
| gi|195150623|ref|XP_002016250.1| | Drosophila persimilis |
| gi|195151905|ref|XP_002016879.1| | Drosophila persimilis |
| gi|195155262|ref|XP_002018524.1| | Drosophila persimilis |
| gi|195166557|ref|XP_002024101.1| | Drosophila persimilis |
| gi|195166559|ref|XP_002024102.1| | Drosophila persimilis |
| gi|195170206|ref|XP_002025904.1| | Drosophila persimilis |
| gi|195328501|ref|XP_002030953.1| | Drosophila sechellia |
| gi|195328503|ref|XP_002030954.1| | Drosophila sechellia |
| gi|195328505|ref|XP_002030955.1| | Drosophila sechellia |
| gi|195333932|ref|XP_002033640.1| | Drosophila sechellia |
| gi|195333934|ref|XP_002033641.1| | Drosophila sechellia |
| gi|195333936|ref|XP_002033642.1| | Drosophila sechellia |
| gi|195335505|ref|XP_002034404.1| | Drosophila sechellia |
| gi|195346295|ref|XP_002039701.1| | Drosophila sechellia |
| gi|195349661|ref|XP_002041361.1| | Drosophila sechellia |
| gi|195352390|ref|XP_002042695.1| | Drosophila sechellia |
| gi|195554937|ref|XP_002076994.1| | Drosophila simulans |
| gi|195574109|ref|XP_002105032.1| | Drosophila simulans |
| gi|195582925|ref|XP_002081276.1| | Drosophila simulans |
| gi|195582927|ref|XP_002081277.1| | Drosophila simulans |
| gi|195582929|ref|XP_002081278.1| | Drosophila simulans |
| gi|195584499|ref|XP_002082042.1| | Drosophila simulans |
| gi|195585290|ref|XP_002082422.1| | Drosophila simulans |
| gi|195591203|ref|XP_002085332.1| | Drosophila simulans |
| gi|195591205|ref|XP_002085333.1| | Drosophila simulans |
| gi|195378564|ref|XP_002048053.1| | Drosophila virilis |
| gi|195379949|ref|XP_002048733.1| | Drosophila virilis |
| gi|195380019|ref|XP_002048768.1| | Drosophila virilis |
| gi|195380021|ref|XP_002048769.1| | Drosophila virilis |
| gi|195380023|ref|XP_002048770.1| | Drosophila virilis |
| gi|195382679|ref|XP_002050057.1| | Drosophila virilis |
| gi|195385268|ref|XP_002051328.1| | Drosophila virilis |
| gi|195385274|ref|XP_002051331.1| | Drosophila virilis |
| gi|195385290|ref|XP_002051339.1| | Drosophila virilis |
| gi|195385296|ref|XP_002051342.1| | Drosophila virilis |
| gi|195389670|ref|XP_002053499.1| | Drosophila virilis |
| gi|195394151|ref|XP_002055709.1| | Drosophila virilis |
| gi|195426451|ref|XP_002061348.1| | Drosophila willistoni |
| gi|195426453|ref|XP_002061349.1| | Drosophila willistoni |
| gi|195426455|ref|XP_002061350.1| | Drosophila willistoni |
| gi|195426459|ref|XP_002061351.1| | Drosophila willistoni |
| gi|195426461|ref|XP_002061352.1| | Drosophila willistoni |
| gi|195426463|ref|XP_002061353.1| | Drosophila willistoni |
| gi|195428437|ref|XP_002062279.1| | Drosophila willistoni |
| gi|195432561|ref|XP_002064285.1| | Drosophila willistoni |
| gi|195441664|ref|XP_002068623.1| | Drosophila willistoni |
| gi|195441666|ref|XP_002068624.1| | Drosophila willistoni |
| gi|195451998|ref|XP_002073168.1| | Drosophila willistoni |
| gi|195455576|ref|XP_002074782.1| | Drosophila willistoni |
| gi|195455695|ref|XP_002074827.1| | Drosophila willistoni |
| gi|195456051|ref|XP_002074981.1| | Drosophila willistoni |
| gi|195478320|ref|XP_002100483.1| | Drosophila yakuba |
| gi|195485150|ref|XP_002090970.1| | Drosophila yakuba |
| gi|195485152|ref|XP_002090971.1| | Drosophila yakuba |
| gi|195485155|ref|XP_002090972.1| | Drosophila yakuba |
| gi|195486646|ref|XP_002091593.1| | Drosophila yakuba |
| gi|195487570|ref|XP_002091964.1| | Drosophila yakuba |
| gi|195488113|ref|XP_002092176.1| | Drosophila yakuba |
| gi|195494695|ref|XP_002094948.1| | Drosophila yakuba |
| gi|195494697|ref|XP_002094949.1| | Drosophila yakuba |
| gi|195504092|ref|XP_002098932.1| | Drosophila yakuba |
| gi|38048495|gb|AAR10150.1| | Drosophila yakuba |
| gi|215504416|gb|EEC13910.1| | Ixodes scapularis |
| gi|215507529|gb|EEC17021.1| | Ixodes scapularis |
| gi|215510058|gb|EEC19511.1| | Ixodes scapularis |
| gi|134801393|emb|CAM35482.1| | Melipona scutellaris |
| gi|63145790|gb|AAY33870.1| | Melipona scutellaris |
| gi|51868671|emb|CAE53465.1| | Mesobuthus caucasicus |
| gi|51868673|emb|CAE53466.1| | Mesobuthus cyprius |
| gi|51868685|emb|CAE53472.1| | Mesobuthus cyprius |
| gi|51868669|emb|CAE53464.1| | Mesobuthus eupeus |
| gi|51868695|emb|CAE53477.1| | Mesobuthus gibbosus |
| gi|51868701|emb|CAE53480.1| | Mesobuthus gibbosus |
| gi|51868709|emb|CAE53484.1| | Mesobuthus gibbosus |
| gi|51868715|emb|CAE53487.1| | Mesobuthus gibbosus |
| gi|51868717|emb|CAE53488.1| | Mesobuthus gibbosus |
| gi|51868723|emb|CAE53491.1| | Mesobuthus gibbosus |
| gi|51868733|emb|CAE53496.1| | Mesobuthus gibbosus |
| gi|51868745|emb|CAE53502.1| | Mesobuthus gibbosus |
| gi|51868755|emb|CAE53507.1| | Mesobuthus gibbosus |
| gi|51868757|emb|CAE53508.1| | Mesobuthus gibbosus |
| gi|51868787|emb|CAE53523.1| | Mesobuthus gibbosus |
| gi|156543378|ref|XP_001599775.1| | Nasonia vitripennis |
| gi|156546882|ref|XP_001606705.1| | Nasonia vitripennis |
| gi|156548112|ref|XP_001606524.1| | Nasonia vitripennis |
| gi|156552049|ref|XP_001603900.1| | Nasonia vitripennis |
| gi|219551908|gb|ACL26692.1| | Nilaparvata lugens |
| gi|212507302|gb|EEB11272.1| | Pediculus humanus corporis |
| gi|212508910|gb|EEB12456.1| | Pediculus humanus corporis |
| gi|21435685|gb|AAM53938.1| | Schistosoma japonicum |
| gi|56753105|gb|AAW24762.1| | Schistosoma japonicum |
| gi|56753521|gb|AAW24964.1| | Schistosoma japonicum |
| gi|56755910|gb|AAW26133.1| | Schistosoma japonicum |
| gi|56756352|gb|AAW26349.1| | Schistosoma japonicum |
| gi|76154396|gb|AAX25885.2| | Schistosoma japonicum |
| gi|21912540|emb|CAD21525.1| | Taenia solium |
| gi|75571591|sp|Q66S17.1| | Natterin |
| gi|75571592|sp|Q66S21.1| | Natterin |
| gi|75571593|sp|Q66S25.1| | Natterin |
| gi|189240579|ref|XP_001814840.1| | Tribolium castaneum |
| gi|198428005|ref|XP_002131710.1| | Ciona intestinalis |
| gi|169847504|ref|XP_001830463.1| | Coprinopsis cinerea okayama7#130 |
| gi|118105776|ref|XP_430295.2| | Gallus gallus |
| gi|134107230|ref|XP_777745.1| | Cryptococcus neoformans var. neoformans B-3501A |
| gi|54293306|ref|YP_125721.1| | Legionella pneumophila str. Lens |
| gi|169859737|ref|XP_001836506.1| | Coprinopsis cinerea okayama7#130 |
| gi|189239558|ref|XP_001816297.1| | Tribolium castaneum |
