# Supplementary material for: Anopheles Gambiae PRS1 Modulates Plasmodium Development at Both Midgut and Salivary Gland Steps
Source: PLoS One. 2010 Jul 12;5(7):e11538. doi: 10.1371/journal.pone.0011538 (PMC2902509; doi:10.1371/journal.pone.0011538)
Supplement: Table S2 — Effects of PRS1 RNAi silencing on Plasmodium development Details of the prevalences and oocyst densities obtained for the four infections after injection of GFP dsRNA or PRS1 dsRNA using P. berghei or P. falciparum, as shown in Fig. 5. (0.08 MB DOC) [file pone.0011538.s002.doc]

| **Tab S2** |  |  |  |  |  |  |
| --- | --- | --- | --- | --- | --- | --- |
|  |  |  |  |  |  |  |
|  | *P. berghei* |  |  |  |  |  |
|  |  | Rep1 | Rep2 | Rep3 | Rep4 | combined p* (meta-analysis) |
|  | Infected/total dsGFP | 24/41 | 22/40 | 7/26 | 14/20 |  |
|  | Infected/total dsPRS1 | 21/41 | 13/40 | 8/28 | 9/17 |  |
|  | Prevalence dsGFP | 0.58 | 0.55 | 0.27 | 0.53 |  |
|  | Prevalence dsPRS1 | 0.51 | 0.32 | 0.29 | 0.47 |  |
| Statistics on prevalence | p (Chi square) | 0.657 | 0.04 | 0.866 | 0.841 | 0.34 |
|  | Median intensity dsGFP | 18.5 | 14.5 | 35 | 27 |  |
|  | Median intensity dsPRS1 | 6 | 5 | 8 | 10 |  |
| Statistics on intensity | p (Mann-Whitney) | 0.070 | 0.037 | 0.054 | 0.083 | **0.004** |
|  |  |  |  |  |  |  |
|  |  |  |  |  |  |  |
|  |  |  |  |  |  |  |
|  | *P. falciparum* |  |  |  |  |  |
|  |  | Rep1 | Rep2 | Rep3 | Rep4 | combined p (meta-analysis) |
|  | Infected/total dsGFP | 35/51 | 9/21 | 21/24 | 23/24 |  |
|  | Infected/total dsPRS1 | 29/51 | 6/21 | 13/24 | 16/24 |  |
|  | Prevalence dsGFP | 0.68 | 0.42 | 0.87 | 0.96 |  |
|  | Prevalence dsPRS1 | 0.56 | 0.28 | 0.54 | 0.66 |  |
| Statistics on prevalence | p (Chi square) | 0.306 | 0.52 | 0.026 | 0.027 | **0.020** |
|  | Median intensity dsGFP | 5 | 2 | 5 | 8 |  |
|  | Median intensity dsPRS1 | 3 | 1 | 10 | 8 |  |
| Statistics on intensity | p (Mann-Whitney) | 0.021 | 0.859 | 0.501 | 0.668 | 0.250 |
|  |  |  |  |  |  |  |
| *: Significant combined p values are indicated in bold | | |  |  |  |  |
|  |  |  |  |  |  |  |
