# Supplementary material for: Anopheles Gambiae PRS1 Modulates Plasmodium Development at Both Midgut and Salivary Gland Steps
Source: PLoS One. 2010 Jul 12;5(7):e11538. doi: 10.1371/journal.pone.0011538 (PMC2902509; doi:10.1371/journal.pone.0011538)
Supplement: Table S3 — Primers used in the study (0.03 MB DOC) [file pone.0011538.s003.doc]

**RT and Q-RT PCR**

*A.gambiae* Ribosomal protein S7 S7-529F CAGCAGACCACCATCGAACA

*A.gambiae* Ribosomal protein S7 S7-647R CTGCTGCAAACTTCGGCTATT

*A.gambiae* PRS1 A15287-318s ACGTCCGATGGGGAGATACT

*A.gambiae* PRS1 A15287-456as GTGTTTTATCGCTCGCACAG

*P. berghei* Cs PbCS-141F TCTCTACTTCCAGGATATGGAC

*P. berghei* Cs PbCS-305R GAGCATCGGCAATAATCTGTTG

*P. falciparum* aldolase pfaldo-1199 ACCCATGGGCTTTAACCTTC

*P. falciparum* aldolase pfaldo-1372 ATTTTCACCACCTGCACCTC

**PRS1 recombinant protein**

*A.gambiae* PRS1 PRSrec5' AAGGAATTCTCCAGGGCCGAAACAAACC

*A.gambiae* PRS1 PRSrec3' ACTCGAGTCGCTCGCACAGCACCTCATAG

**ds RNA synthesis**

*A.gambiae* PRS1 T7PRS5'b GAATTGTAATACGACTCACTATAGGGCTGTTCATACGAAGTGCTT

*A.gambiae* PRS1 T7PRS3'b GAATTGTAATACGACTCACTATAGGGACACTTCCGCACCACCGTA

*A.gambiae* TCL4 T7-TCL4-5' GAATTGTAATACGACTCACTATAGGGATTACCCTCGTTTGTCTAAT

*A.gambiae* TCL4 T7-TCL4-3' GAATTGTAATACGACTCACTATAGGGCAGAAACATTCGAGGAGTT

GFP T7-GFP GAATTGTAATACGACTCACTATAGGGCATGGTGAGCAAGGGCGA

GFP T7-GFP GAATTGTAATACGACTCACTATAGGGCTTACTTGTACAGCTCGTC
