# Supplementary figures and images for: Anopheles Gambiae PRS1 Modulates Plasmodium Development at Both Midgut and Salivary Gland Steps
Source: PLoS One. 2010 Jul 12;5(7):e11538. doi: 10.1371/journal.pone.0011538 (PMC2902509; doi:10.1371/journal.pone.0011538)

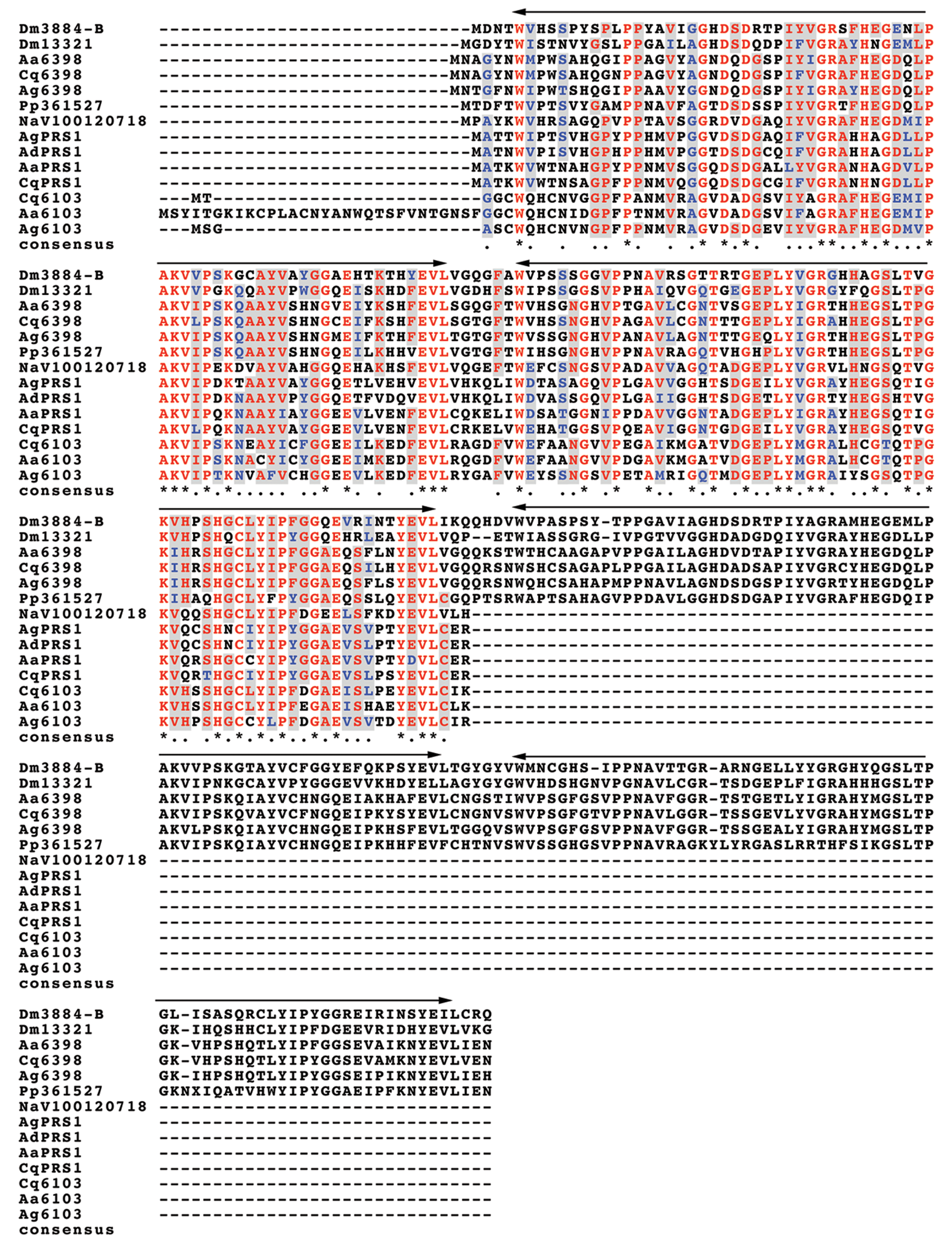

Supplement: Figure S1 — Alignment of DM9-containing proteins belonging to PRS1 subfamily. DM9 proteins whose genes are located on chromosome 2 in An. gambiae and their closest homologues in An. gambiae (Ag), An. darlingi, Culex quinquefasciatus (Cq), A. aegypti (Aa), Phlebotomus papatasi (Pp) and Nasonnia vitripennis (NaV) were aligned together with two DM9 proteins from Drosophila (Dm: D. melanogaster) and this alignment was used for the tree shown in Fig. 1. The complete names of the proteins and their accession numbers are given in Tab. S1. Identical or similar residues found in more than 60% of protein sequences are highlighted in red and blue, respectively. DM9 motifs are indicated by double-ended arrows (4.57 MB TIF) [file pone.0011538.s004.tif]

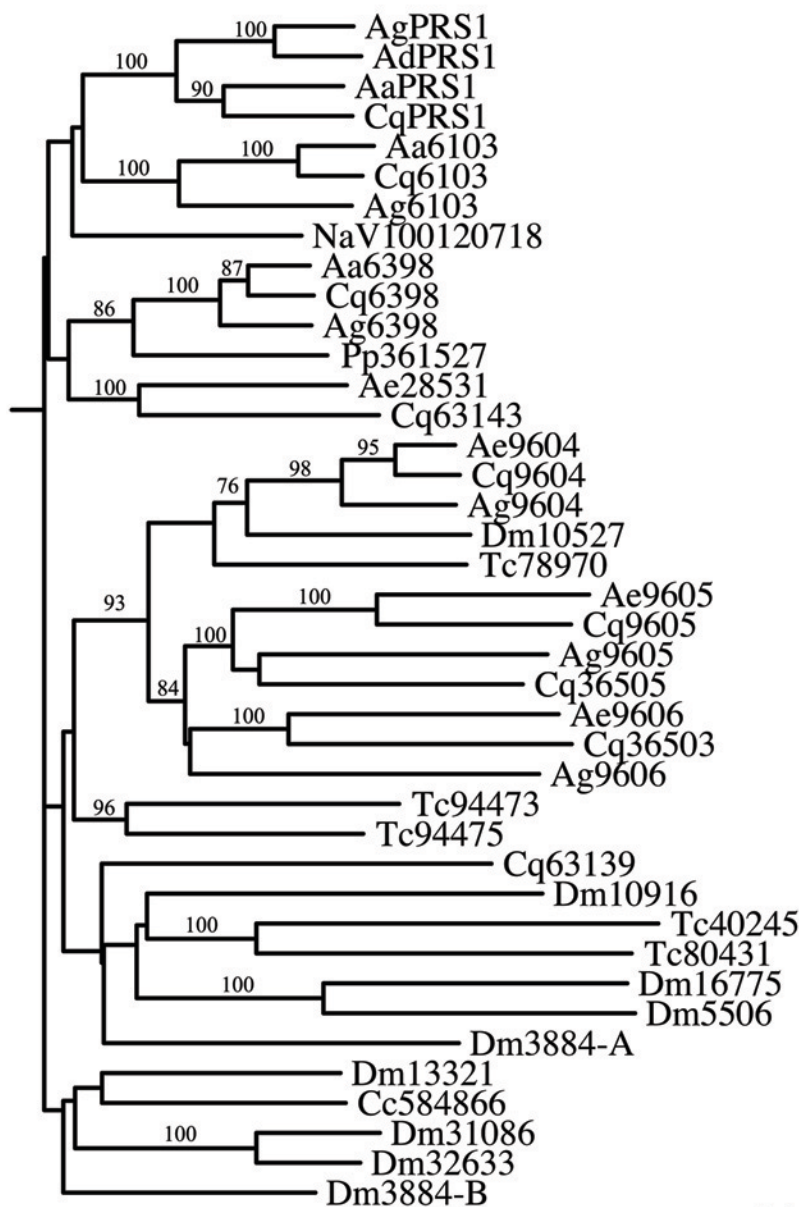

Supplement: Figure S2 — Phylogenetic tree showing the relationships between in DM9 proteins found in An. gambiae (Ag), An. darlingi (Ad), Culex quinquefasciatus (Cq), A. aegypti (Aa), Phlebotomus papatasi (Pp), Nasonnia vitripennis (NaV) and D. melanogaster (Dm). The tree is unrooted. Bootstrap values superior to 75 per cent are indicated. Scale bar represents 10% differences in protein sequences. (0.97 MB PDF) [file pone.0011538.s005.pdf]

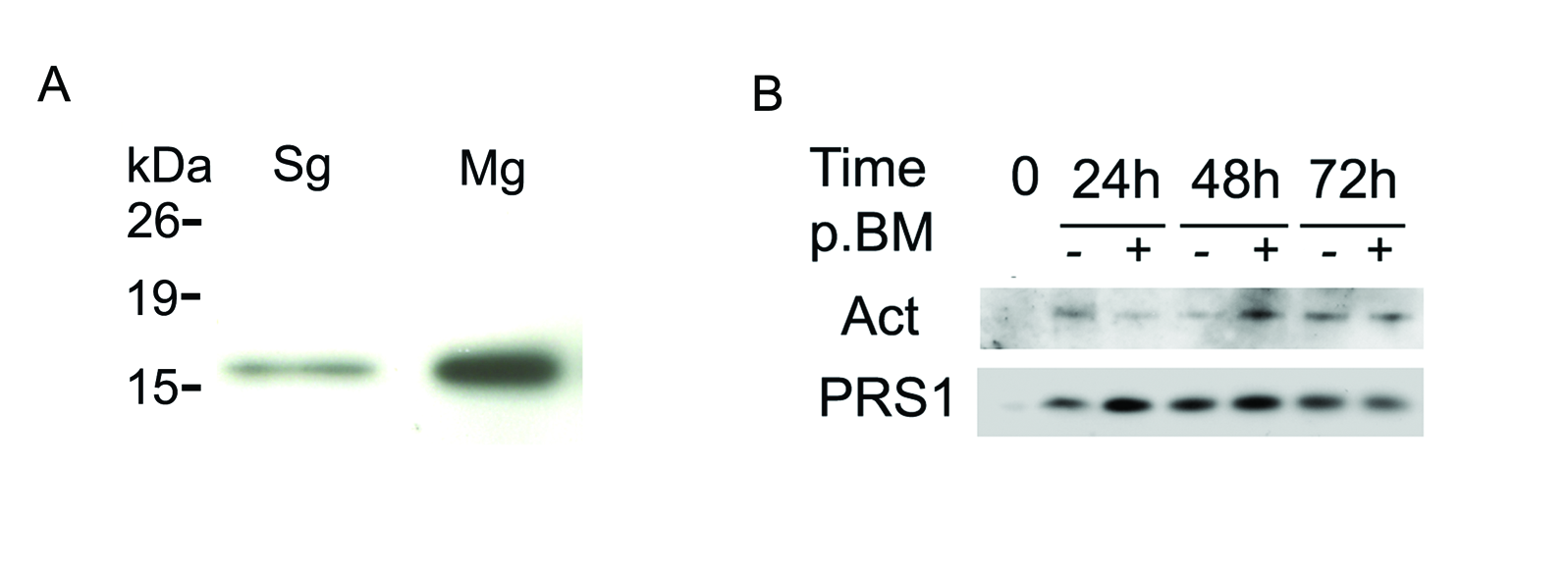

Supplement: Figure S3 — Immunoblots of midgut and salivary gland extracts using anti-PRS1 antibodies. Molecular masses of the markers are indicated in kDa. A. Immunoblot of midgut and salivary gland extracts from non-infected An. gambiae. B. Immunoblot of An. gambiae midgut sheets before (time 0) or at various times (24 h, 48 h, 72 h) after an infected (+) or a non-infected (−) BM. (3.76 MB TIF) [file pone.0011538.s006.tif]

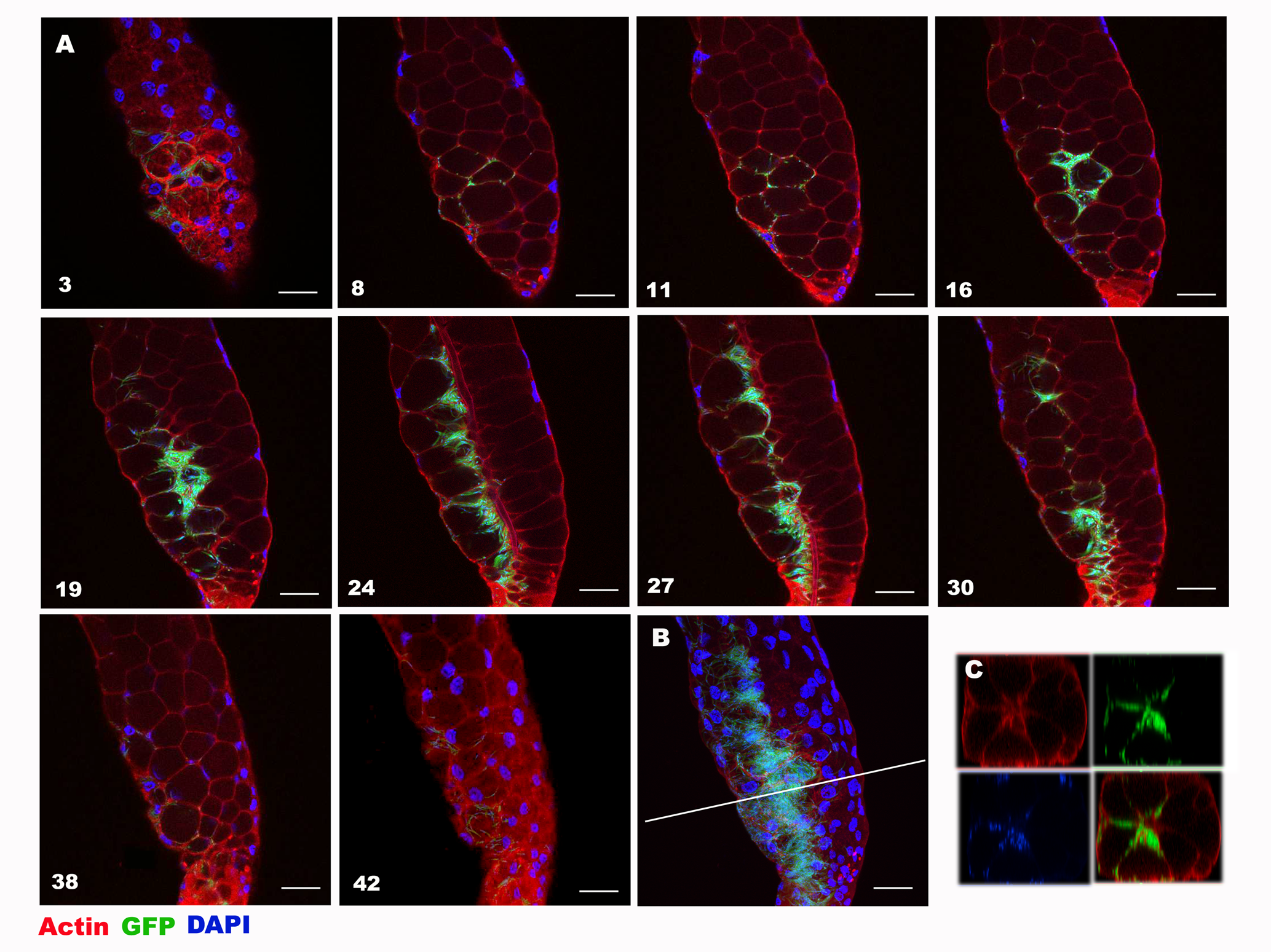

Supplement: Figure S4 — Phalloidin labeling of the actin network in salivary glands. A: Focal sections from upper to lower sections showing merge labeling for actin (red), GFP (green) and DAPI (blue). The number in the lower left corner indicates the number of the section. Bar: 20 µm. B: Z stack projection of all the sections. The white line indicates the direction of the plane used in C for 3-D reconstruction. C: 3-D reconstruction of a cross-section of the gland according to the direction defined by the white line in B. Note that sporozoites are localized in close proximity to the actin network. (3.27 MB TIF) [file pone.0011538.s007.tif]

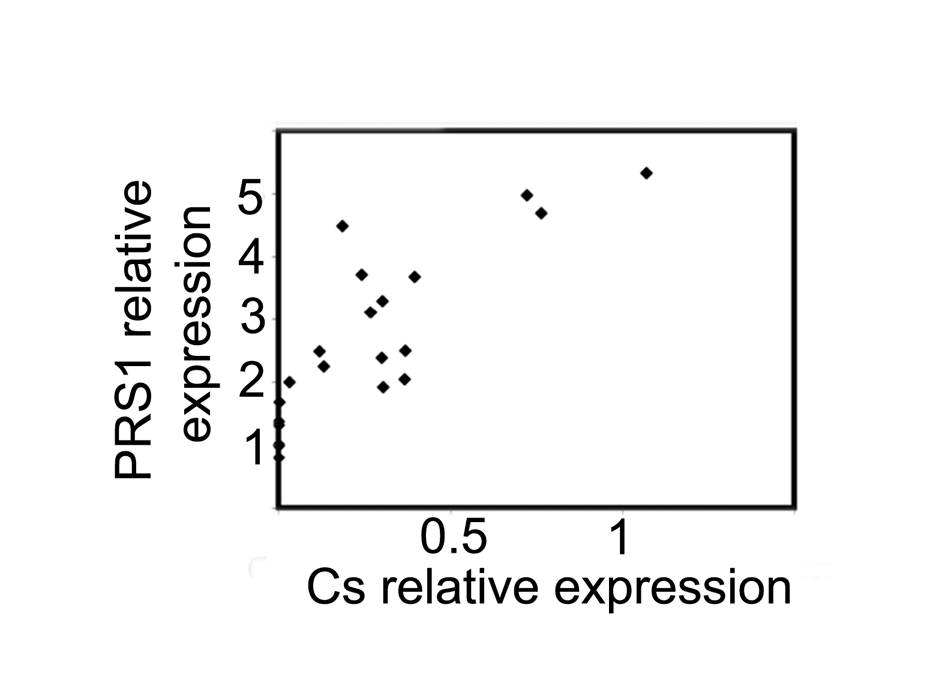

Supplement: Figure S5 — Correlation between PRS1 and Cs expression in salivary glands; mRNAs for PRS1 and CS were quantified by qRT-PCR in different preparations of salivary glands after invasion by P. berghei. CS expression is used as a marker of the number of sporozoites inside the glands. A correlation between both sets of data is demonstrated by the Pearson correlation coefficient (R = 0.84; R2 = 0.7, p<0.001). (2.00 MB TIF) [file pone.0011538.s008.tif]
